# Recurrent Copy Number Variants and Psychiatric Outcomes in the Context of Polygenic Scores

**DOI:** 10.1001/jamapsychiatry.2026.1064

**Published:** 2026-05-27

**Authors:** Morteza Vaez, Simone Montalbano, Ryan Waples, Morten Dybdahl Krebs, Kajsa-Lotta Georgii Hellberg, Jesper Gådin, Daniel Stow, Peter Holmans, Marianne van den Bree, Anders D. Børglum, Dorte Helenius, Thomas Werge, Andrew J. Schork, Andrés Ingason

**Affiliations:** 1 Institute of Biological Psychiatry, Mental Health Center Sct Hans, Amager-Hvidovre Hospital, Copenhagen University Hospital, Roskilde, Denmark; 2 The Lundbeck Foundation Initiative for Integrative Psychiatric Research (iPSYCH), Copenhagen and Aarhus, Denmark; 3 Wolfson Institute for Population Health, Queen Mary University of London, Charterhouse Square, London, United Kingdom; 4 Centre for Neuropsychiatric Genetics and Genomics & Neuroscience and Mental Health Innovation Institute, Division of Psychological Medicine and Clinical Neurosciences, Cardiff University, Cardiff, United Kingdom; 5 Department of Biomedicine—Human Genetics and the iSEQ Center, Aarhus University, Aarhus, Denmark; 6 Center for Genomics and Personalized Medicine, Aarhus, Denmark; 7 Department of Clinical Medicine, University of Copenhagen, Copenhagen, Denmark; 8 Center of Public Health Sciences, Faculty of Medicine, University of Iceland, Reykjavík, Iceland

## Abstract

**Importance:**

Although both recurrent copy number variants (rCNVs) and polygenic scores (PGSs) impart risk for psychiatric disorders, it remains unclear how they contribute jointly to this risk.

**Objective:**

To estimate and compare absolute risk of psychiatric disorders associated with rCNVs and PGSs, independently and jointly.

**Design, Setting, and Participants:**

This genetic association study applied data from the Lundbeck Foundation Initiative for Integrative Psychiatric Research (iPSYCH) case-cohort sample of individuals born in Denmark (1981-2008) and followed up until 2015, including all individuals with a hospital diagnosis of attention-deficit/hyperactivity disorder (ADHD), autism spectrum disorder (ASD), schizophrenia spectrum disorder (SSD), or major depressive disorder (MDD), and a subcohort randomly drawn from the source population. Data were analyzed from September 2023 to May 2025.

**Exposures:**

Carrier status was determined at 27 autosomal rCNV loci and PGSs for psychiatric (and other) outcomes from neonatal blood samples genotyped on microarrays and summary statistics from published association studies.

**Main Outcomes and Measures:**

Absolute risks were estimated for ADHD, ASD, MDD, and SSD during follow-up using a weighted survival analysis framework, and joint effects of rCNV carriage and PGSs were assessed by fitting generalized linear models.

**Results:**

In 94 276 unrelated European-ancestry individuals (mean [SD] age at follow-up, 21.9 [7.0] years; 50 653 male [53.7%]), rCNV carriage was associated with increased risk of ASD, ADHD, and SSD but not MDD (β = 0.33; 95% CI, 0.27-0.39; β = 0.29; 95% CI, 0.23-0.35; β = 0.25; 95% CI, 0.17-0.33; and β = 0.04; 95% CI, −0.03 to 0.11, respectively); each PGS was positively associated with risk of the corresponding disorder (β = 0.14; 95% CI, 0.12-0.16; β = 0.28; 95% CI, 0.26-0.30; β = 0.28; 95% CI, 0.26-0.30; and β = 0.38; 95% CI, 0.36-0.40, respectively). PGSs identified more individuals than rCNV carriage at comparable levels of absolute risk, except for ASD. A negative interaction was observed between 16p13.11 duplication and ADHD-PGS on ADHD risk (β = −0.51; 95% CI, −0.86 to −0.16), and there was a trend toward negative rCNV-PGS interaction coefficients across aggregated rCNV groups and each of the 9 most common rCNVs (27 of 39 tests, *P* binomial = .01).

**Conclusions and Relevance:**

Findings of this genetic association study highlight the complementary value of rCNVs and PGSs for risk assessment in psychiatric disorders, with indications that PGSs can stratify risk among medium- and high-impact rCNV carriers and rCNV-associated risk may, in some instances, be attenuated among individuals with low PGSs.

## Key Points

**Question:**

How do recurrent copy number variants (rCNVs) and polygenic scores (PGSs) compare and interact as genetic risk factors for psychiatric disorders in the population?

**Findings:**

In a large population-based genetic association study of 94 276 participants, both PGSs and rCNVs were associated with absolute risk of psychiatric disorders, with PGSs identifying at-risk individuals at a higher rate for attention-deficit/hyperactivity disorder, depression, and schizophrenia but not autism. There was no consistent statistical evidence of interactions between rCNVs and PGSs.

**Meaning:**

Findings suggest that PGSs and rCNVs play broadly complementary roles in risk prediction in psychiatry, with PGSs being able to stratify risk among carriers of medium- and high-impact rCNVs.

## Teaser

This genetic association study evaluates the association of recurrent copy number variants and polygenic scores with genetic risk factors for psychiatric disorders in the population.

## Introduction

Recurrent copy number variants (rCNVs) are considered to include some of the strongest individual predictors of psychiatric outcomes,^1,2,3^ making them a focus for basic and translational research. Early case-control studies indicated near-complete penetrance of certain rCNVs, such as the 22q11.2 deletion, during a period when few alternative explanations existed for the high familiality of psychiatric disorders.^3,4,5^ Although penetrance estimates for 22q11.2 deletion and other rCNVs have since come down with larger, more population-representative studies,^6,7,8^ rCNVs remain important in clinical applications, particularly for children with congenital malformations, neurodevelopmental disorders, and autism spectrum disorder (ASD).^9,10^ Nevertheless, the rarity of rCNVs limits their explanatory power at the population level, and recent estimates suggest their effect sizes are broadly comparable to those of other genetic risk factors such as family history and polygenic burden.^8,11,12,13,14^

Genome-wide association studies (GWAS) in psychiatry have identified hundreds of associated variants and revealed a substantial polygenic component, comprising the cumulative effects of thousands of small-effect common variants across the genome.^15,16,17,18^ Polygenic scores (PGSs) use these aggregated effects to estimate an individual’s genetic liability, with demonstrated predictive power across diverse traits, including psychiatric outcomes.^19,20,21,22^ Although clinical applications of PGSs have progressed in cardiology^20,23^ and oncology,^22^ its use in psychiatry remains largely exploratory. Notably, recent studies^20,24,25,26,27^ of cardiometabolic disorders, breast cancer, and depression suggest that PGSs can approximate and modulate the effects of rare high-impact variants. Similarly, animal models have long demonstrated that polygenic background can shape phenotypic expression of high-impact gene variants.^28,29^ These findings underscore the importance of integrating PGSs and rCNVs for genetic risk assessment in psychiatry.

Precision medicine and individualized treatment strategies will demand a comprehensive understanding of risk that considers multiple measures and markers. This implies not only estimating the effects of rCNVs accurately in a population but also assessing their synergy with other risk factors that may moderate or compensate for risk. To date, only a few studies have investigated the joint contribution of rCNVs and PGSs to psychiatric risk, and thus, our understanding remains incomplete and limited in scope.^30,31,32,33,34,35^ Case-control studies in schizophrenia^31,32,34^ and attention-deficit/hyperactivity disorder (ADHD)^30^ have reported a lower polygenic burden among affected rCNV carriers, consistent with both common and rare variants contributing toward a similar liability threshold for the disorders.^36^ In contrast, a study of major depressive disorder (MDD) did not identify differences in PGSs among affected rCNV carriers,^37^ and to our knowledge, no study to date has examined this association in ASD. Moreover, previous investigations were often limited by small sample sizes, nonrepresentative populations, focus on specific rCNVs, and a lack of clinically interpretable measures of absolute risk.^30,31,32,33,34,37^

Here, we use the population-based Lundbeck Foundation Initiative for Integrative Psychiatric Research (iPSYCH2015) case-cohort study^38^ to (1) estimate the absolute risk associated with rCNVs and PGSs, independently and jointly; (2) quantify the proportion of the population whose PGS burden confers risk equivalent to that conferred by rCNVs; and (3) compare the distribution of multiple PGSs among rCNV carriers and noncarriers to explore etiological heterogeneity among cases.

## Methods

### Study Design

In accordance with Danish legislation, the Danish Scientific Ethics Committee waived the requirement for informed consent for the iPSYCH project, including construction of the iPSYCH2015 case-cohort sample. This analysis was conducted on data stored on the secure GenomeDK high-performance computing facility at the Aarhus Genome Data Center. This study followed the Strengthening the Reporting of Genetic Association Studies (STREGA) reporting guidelines.

iPSYCH2015 is a case-cohort study of 140 116 individuals sampled from 1 657 449 singleton births in Denmark between May 1, 1981, and December 31, 2008.^38^ The case sample comprises 92 531 individuals with registered hospital diagnoses, including MDD (*International Statistical Classification of Diseases and Related Health Problems, Tenth Revision *[*ICD-10*]: F32-F33; n = 37 555), ASD (*ICD-10*: F84; n = 24 975), bipolar disorder (*ICD-10*: F30-F31; n = 3819), schizophrenia spectrum disorder (SSD; *ICD-10*: F20-F29; n = 16 008), schizophrenia (*ICD-10*: F20; n = 8113), and ADHD (*ICD-10*: F90; n = 29 668). The subcohort includes 50 615 individuals randomly selected from the birth cohort.^38^ Diagnostic codes were available until December 31, 2015, and obtained from the Psychiatric Central Research Register.^38^ For this study, we did not include individuals ascertained to have bipolar disorder as the number of bipolar disorder cases is insufficient for analysis of rare exposures. Information on self-reported race and ethnicity is not collected in the Danish national registries that the study is based on and is, therefore, not available.

DNA was extracted from dried blood spots stored in the National Neonatal Screening Biobank.^39^ Genotyping was performed on Infinium Psych Chip, version 1.0, or Global Screening Array, version 2.0 (Illumina). GenomeStudio software (Illumina) was used to extract B-allele frequency (BAF) and probe intensities (log-R Ratio; LRR). Further details regarding the quality control and genotyping call rate are described elsewhere.^38^ Genotyped samples on both arrays were first phased and imputed separately using BEAGLE 5.4^40,41^ and then merged (eMethods in Supplement 1).

Unrelated individuals of broadly northern European ancestry were identified based on genetic principal components (PCs) and robust kinship inference approaches in the computer program KING^42^ (eMethods in Supplement 1).

### CNV Calling

We included 54 rCNVs from a previous study of the iPSYCH2015.^8^ Briefly, CNV calling was performed by PennCNV.^43^ Samples with outlying LRR-SD, BAF-drift, and/or guanine-cytosine–wave factor values were removed from further analysis, and PennCNV calls in the remaining samples were validated using the QCtreeCNV pipeline.^44^ Additional details are described elsewhere.^8^ Locuswide loss of function observed/expected upper fraction (LOEUF) score of each locus was derived by summing the inverted LOEUF (invLOEUF) scores of all genes overlapping at least 50% with the locus, obtained from the GnomAD database, version 2.1.1^45^ as described elsewhere.^8^

### PGS Generation

PGSs were generated using the score module in PLINK, version 2.00a2.3,^46^ after rescaling single-nucleotide polymorphism effects from external GWAS using SbayesR.^47^ Scores were standardized to the mean and variance of the population subcohort using *z*-score transformation (eMethods in Supplement 1).

### Statistical Analysis

We used weighted survival models from the survival package in R^48^ to estimate absolute risks with age at first diagnosis as the outcome and rCNV or PGS group status as individual and joint predictors. Absolute risk curves were smoothed using the cobs function from the cobs R package^49^ to ensure increments did not indicate individual cases (R Project for Statistical Computing) (eMethods in Supplement 1).

We compared PGS- and rCNV-derived risk by first estimating population absolute risk using weighted survival models and deriving PGS effect estimates for each outcome using covariate-adjusted logistic regression. We then generated an artificial PGS distribution by direct sampling of 100 000 representative quantiles based on the mean and SD observed in the random subcohort. For each quantile, we calculated the expected absolute risk using the estimated population risk and PGS-derived risk ratios. Finally, we determined the proportion of individuals whose PGS-derived absolute risk exceeded the rCNV-associated absolute risk, by evaluating the upper tail of the PGS distribution (eMethods in Supplement 1).

To investigate independent and combined effects of rCNVs and PGSs on outcomes, we fitted a base logistic GLM with age, sex, and genotyping array as covariates. rCNV, PGS, and their interaction were then added sequentially and compared using a likelihood ratio test (LRT). To correct for population stratification, PGSs were residualized for 20 genetic PCs from unrelated European individuals.

We compared the mean PGS of cases and controls that were rCNV carriers and noncarriers in logistic and linear regression models with rCNV status as the outcome. Nested models included age, sex, genotyping array, and 20 PCs; full models added the corresponding disorder-specific PGS. Model fit was assessed using LRTs. To investigate broader phenotypic profiles, simple and multinomial logistic regressions were applied within each psychiatric outcome, comparing rCNV carriers and noncarriers. Base models used the same covariates, followed by sequential addition of psychiatric, behavioral, and somatic PGSs, with LRTs used to evaluate model improvement (eMethods in Supplement 1).

## Results

After genotyping and quality control, 94 276 unrelated individuals (mean [SD] age at follow-up, 21.9 [7.0] years; 43 623 female [46.3%]; 50 653 male [53.7%]) of European ancestry remained (59 567 cases and 37 001 in the population subcohort, including an overlap of 2209 cases in the subcohort). Age at end of follow-up ranged between 7.0 and 34.6 years. Given their rarity, we grouped rCNVs in several ways according to expected impact (eFigure 1 in Supplement 1). On a comparison revealing similar pattern of impact stratification, we opted for the summed invLOEUF score as the most biologically objective measure to group carriers: low impact (<10), moderate impact (10-25), and high impact (>25) (eFigure 1, eTable 1, and eTable 6 in Supplement 1 and eTables 2-5 in Supplement 2).

### rCNVs and PGSs and Absolute Risk of Psychiatric Disorders

**Figure 1.**
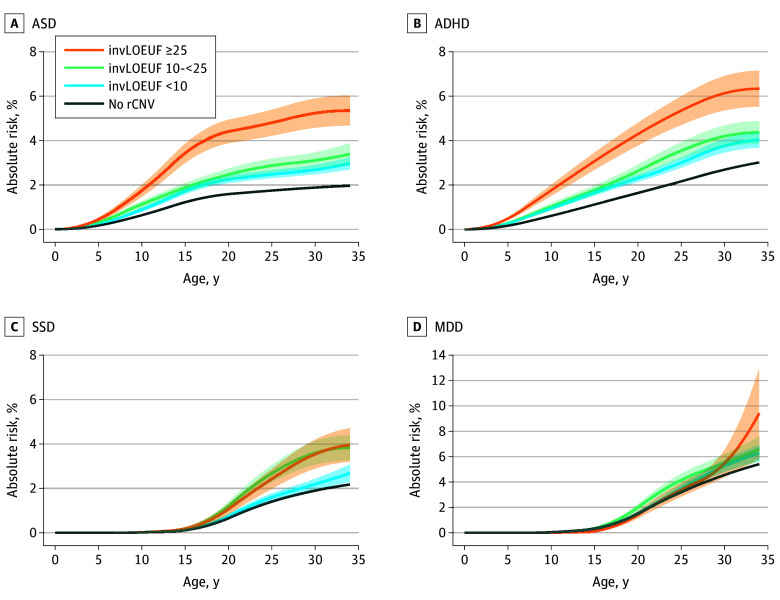
Line Graphs Depicting Absolute Risk of Psychiatric Disorders Associated With Recurrent Copy Number Variant (rCNV) Groups in the Lundbeck Foundation Initiative for Integrative Psychiatric Research (iPSYCH2015) A, Autism spectrum disorder (ASD). B, Attention-deficit/hyperactivity disorder (ADHD). C, Schizophrenia spectrum disorder (SSD). D, Major depressive disorder (MDD). Absolute risks associated with rCNV groups were derived by fitting the surv.fit function using the survival package in R for each disorder separately. rCNVs were categorized into 3 groups based on their corresponding locus’s inverted loss of function observed/expected upper fraction (invLOEUF) score. Shading represents the SEs of each rCNV group. The MDD panel is displayed on a different y-axis scale than the other disorders. All the curves are smoothed by the cobs function from the cobs package in R (R Project for Statistical Computing).

For ASD, absolute risk at the end of follow-up ranged from 2.0% for noncarriers to 5.4% for high-impact rCNV carriers. For ADHD, SSD, and MDD, the corresponding estimates ranged from 3.0% to 6.3%, 2.2% to 4.0%, and 5.4% to 9.4%, respectively. The GLM tests for increased risk of diagnosis with carriage of rCNVs with increasing invLOEUF score found positive associations observed for ASD (β = 0.33; 95% CI, 0.27-0.39; *P* < 2.2 × 10^−16^), ADHD (β = 0.29; 95% CI, 0.23-0.35; *P* < 2.2 × 10^−16^), and SSD (β = 0.25; 95% CI, 0.17-0.33; *P* = 5.66 × 10^−10^), but not MDD (β = 0.04; 95% CI, −0.03 to 0.11; *P* = .27) (Figure 1 and eFigure 2 and eTable 7 in Supplement 1).

**Figure 2.**
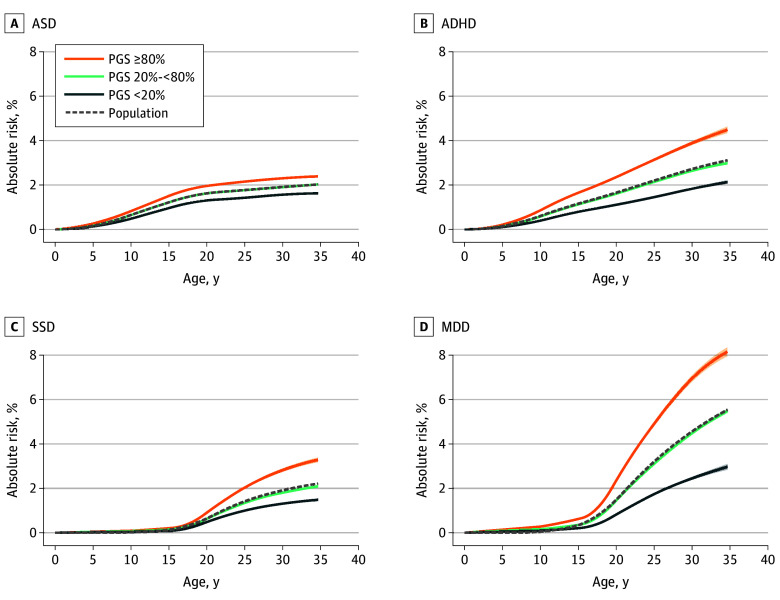
Line Graphs Depicting Absolute Risk of Psychiatric Disorders Associated With Polygenic Score (PGS) Groups in the Lundbeck Foundation Initiative for Integrative Psychiatric Research (iPSYCH2015) A, Autism spectrum disorder (ASD). B, Attention-deficit/hyperactivity disorder (ADHD). C, Schizophrenia spectrum disorder (SSD). D, Major depressive disorder (MDD). Absolute risks associated with PGS groups were derived by fitting the surv.fit function using the survival package in R for each disorder separately. Individuals were categorized into 3 groups based on their PGSs for each corresponding disorder. Shading represents the SEs of each PGS group. The dashed line indicates the absolute risk of the corresponding disorder within the average population. All the curves are smoothed by the cobs function from the cobs package in R (R Project for Statistical Computing).

To examine the association between PGS and absolute risk of psychiatric disorders, we stratified individuals based on PGS percentiles; low (<20th), medium (20th-80th), and high (>80th). For ASD, absolute risk at the end of follow-up increased from 1.6% to 2.4% from the low to high PGS group, and for ADHD, SSD, and MDD, the corresponding risk estimates increased from 2.1% to 4.5%, 1.5% to 3.3%, and 3.0% to 8.1%, respectively (Figure 2 and eFigure 3 in Supplement 1). For all disorders, PGS was significantly associated (*P* < 3.88 × 10^−45^) with increased risk of diagnosis in a GLM test (ASD: β = 0.14; 95% CI, 0.12-0.16; ADHD: β = 0.28; 95% CI, 0.26-0.30; SSD: β = 0.28; 95% CI, 0.26-0.30; and MDD: β = 0.38; 95% CI, 0.36-0.40, respectively) (eTable 7 in Supplement 1).

**Figure 3.**
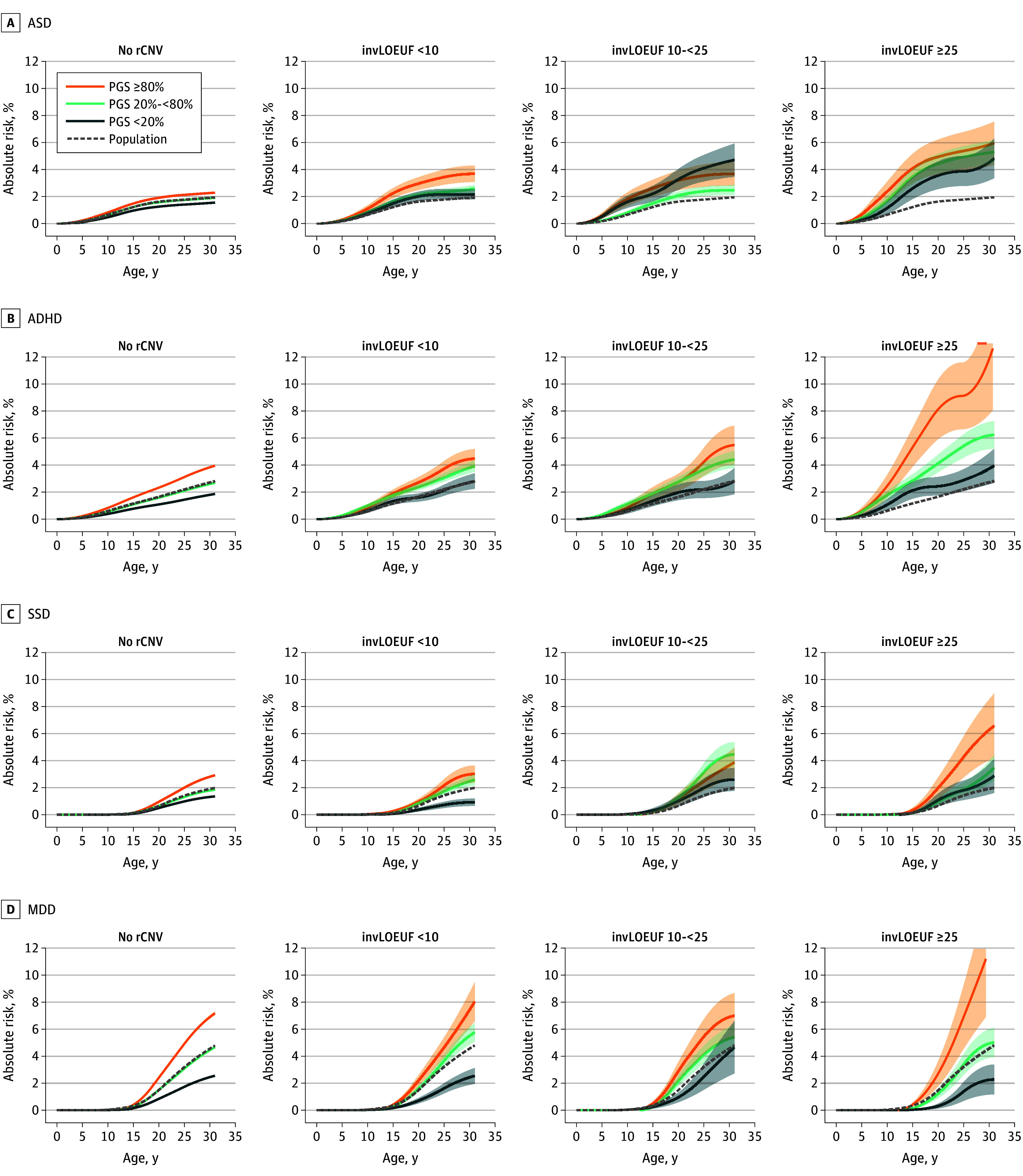
Line Graphs Depicting Absolute Risk of Psychiatric Disorders Associated With Recurrent Copy Number Variant (rCNV) and Polygenic Score (PGS) Groups Combined in the Lundbeck Foundation Initiative for Integrative Psychiatric Research (iPSYCH2015) Joint absolute risks associated with rCNV and PGS group were derived by fitting the surv.fit function using the survival package in R for each disorder separately. For enhancing the visualization, absolute risk curves associated with each disorder are shown for noncarriers and carriers within each of the rCNV groups separately in 4 different panels, stratified by the PGS groups. Shading represents the SEs of each PGS group. Due to low carrier counts across the strata at the follow-up age older than 31 years, absolute risk curves are shown until the follow-up age of 31 years. The dashed line indicates the absolute risk of the corresponding disorder within the average population. All the curves are smoothed by the cobs function from the cobs package^49^ in R (R Project for Statistical Computing). invLOEUF indicates inverted loss of function observed/expected upper fraction.

**Figure 4.**
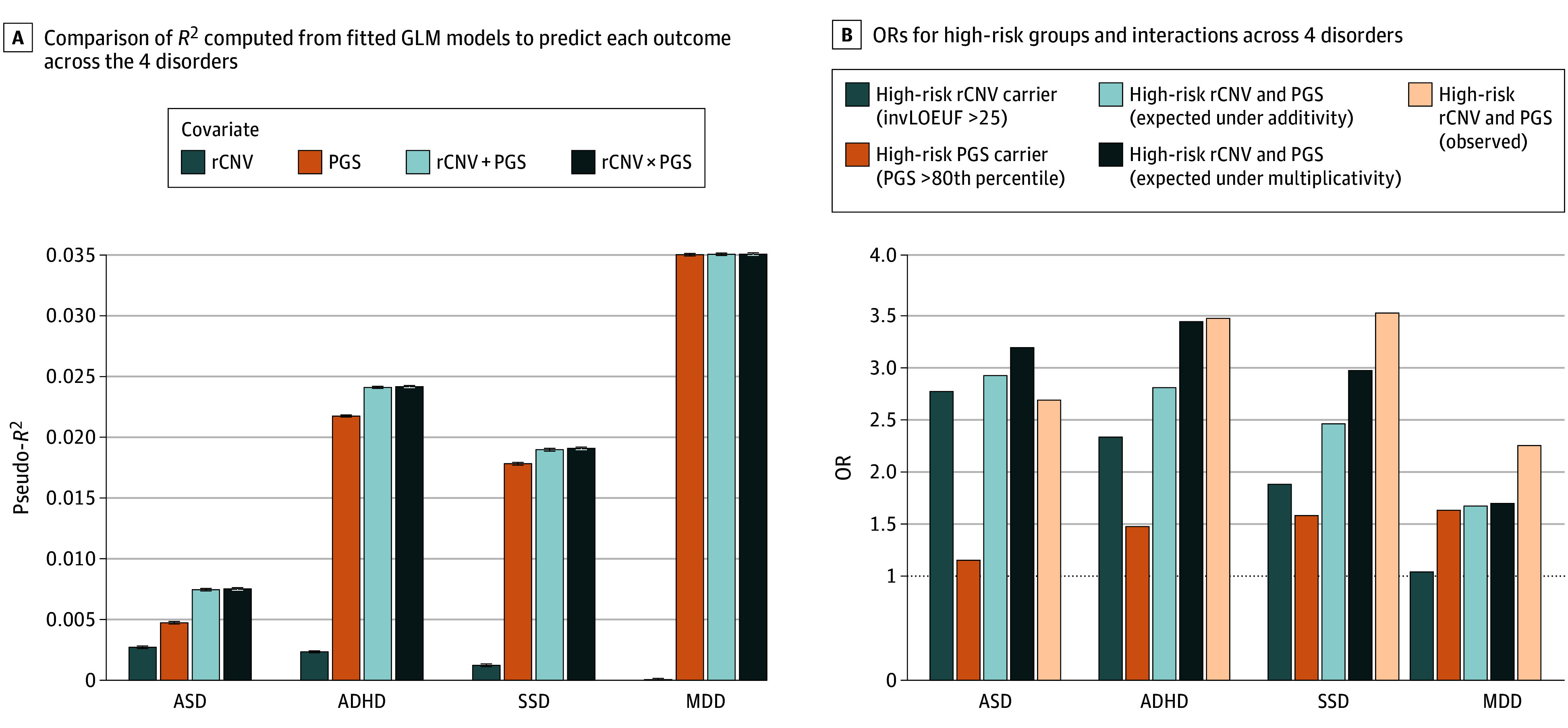
Bar Graphs Showing the Comparison of Variance Explained and Odds Ratios (ORs) Attributed to Recurrent Copy Number Variant (rCNV) and Polygenic Score (PGS) Groups Derived From Fitted Generalized Linear Models (GLMs) for Psychiatric Disorders in the Lundbeck Foundation Initiative for Integrative Psychiatric Research (iPSYCH2015) A, Comparison of Nagelkerke *R^2^* computed from the fitted GLM models to predict each outcome across the 4 disorders. Formal comparisons between models were performed by likelihood ratio tests (LRTs), which form the basis for all statistical conclusions regarding the contributions of rCNVs and PGSs, and Nagelkerke *R^2^* values were primarily used to illustrate an approximate measure of variance explained by each genetic predictor. Nagelkerke *R^2^* was computed for each model using the PseudoR2 function from the DescTools package. Each bar indicates *R^2^* values by different colors that are derived from fitted GLMs using rCNV and PGS status individually, as well as additive (rCNV+PGS) and interactive (rCNV × PGS) forms for predicting each diagnosis, respectively. Error bars indicate SEs of each Nagelkerke *R^2^*. B, ORs for the high-risk rCNV carrier group (inverted loss of function observed/expected upper fraction [invLOEUF] >25), the high-risk PGS carrier group (PGS >80th percentile), and their interactions across 4 disorders, distinguished by color. GLM analyses testing multiplicative interactions between rCNV-invLOEUF and PGS groups did not yield significant results. To illustrate potential joint effects, we additionally fitted GLMs to predict each diagnosis and derived ORs under additive and multiplicative assumptions, as well as for the observed interaction. Expected ORs were calculated by first estimating ORs for each genetic factor separately (high-risk rCNV carrier, high-risk PGS carrier) and then combining them by summation (additive) or multiplication (multiplicative). Observed interaction ORs were obtained by summing the effects of high-risk rCNVs, high-risk PGSs, and their interaction term from GLMs including the interaction. In the fitted models, the reference groups were individuals without rCNVs and those with PGS percentiles between the 20th and 80th (eMethods in Supplement 1).

When fitted jointly, risk broadly increased both with higher PGS and more impactful rCNVs across all disorders (Figure 3 and eFigure 4 in Supplement 1); although for SSD, no rCNV group showed a noticeable increase in risk over noncarriers in the low PGS strata, suggesting that PGS may, in some cases, be associated with a reduction in the impact of rCNVs. However, although fitted GLMs revealed jointly significant effects of PGS and rCNV groups on the risk of all disorders (*P* < 5.75 × 10^−8^) except MDD, corresponding tests for statistical interaction on the log-odds scale between PGS and rCNV were not significant (Figure 4A, eTable 8 in Supplement 2, and eTables 9 and 10 in Supplement 1). We also found no significant interaction on the log-odds scale between PGSs and individual rCNVs, except for the 16p13.11 duplication and ADHD-PGS (β = −0.51; 95% CI, −0.86 to −0.16; *P* = .006), although, there was a significant trend for negative interaction coefficients (27 of 39; *P* = .01 in a binomial test) (eTables 11 and 12 in Supplement 2 and eTables 13 and 14 in Supplement 1). For illustrative purposes, we depict interaction effect estimates on additive and multiplicative scales in Figure 4B.

### Comparison of the Proportion of Individuals With Polygenic Risk Exceeding rCNV-Associated Risk Levels

**Figure 5.**
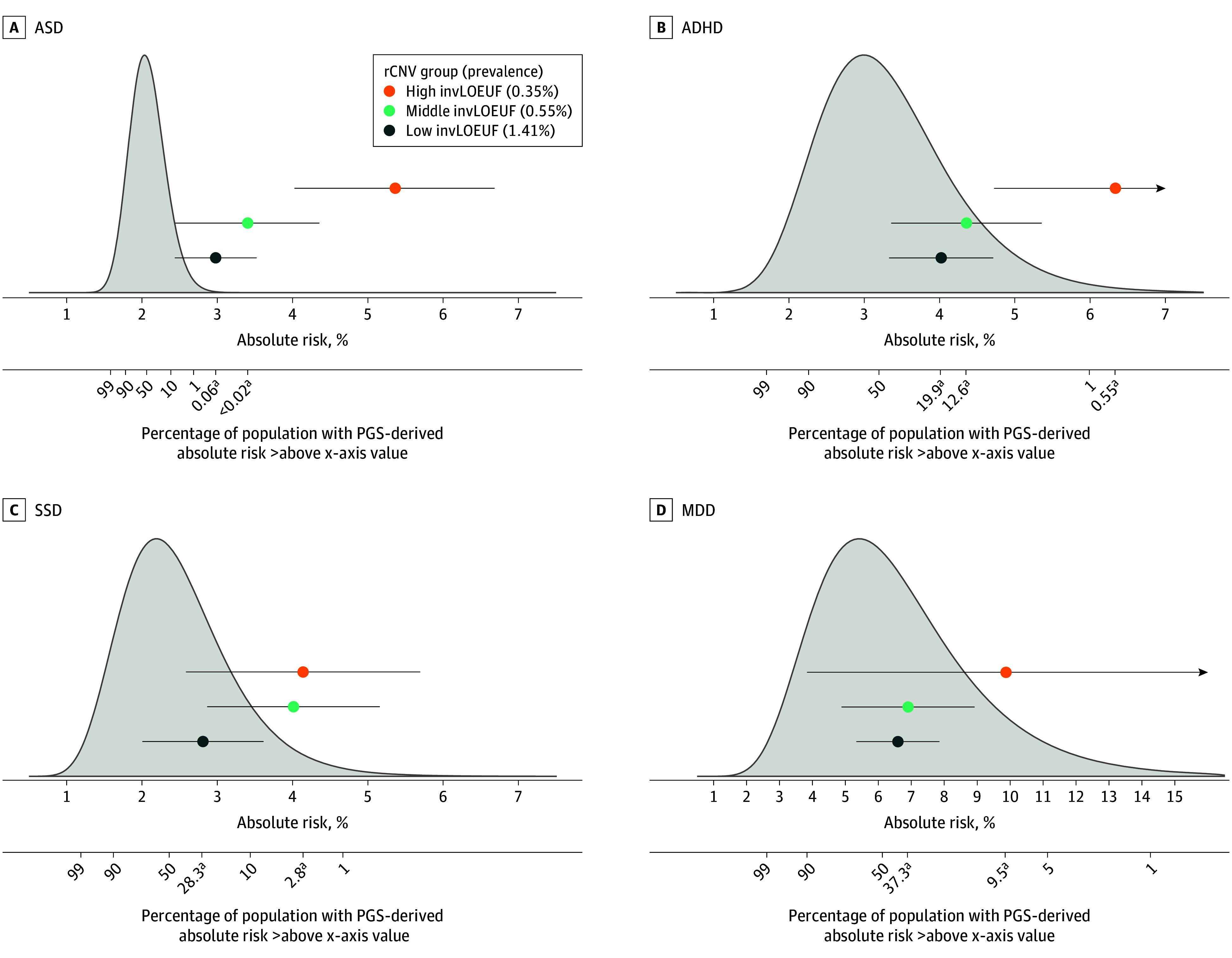
Density Plots and Point Estimates Showing the Comparison of Absolute Risk Estimates and Proportion of At-Risk Individuals Attributed to Recurrent Copy Number Variants (rCNVs) and Polygenic Scores (PGSs) for Psychiatric Disorders in the Lundbeck Foundation Initiative for Integrative Psychiatric Research (iPSYCH2015) Random Cohort A, Autism spectrum disorder (ASD). B, Attention-deficit/hyperactivity disorder (ADHD). C, Schizophrenia spectrum disorder (SSD). D, Major depressive disorder (MDD). Each panel shows the comparison between absolute risks attributed to rCNV groups at the end of follow-up and those derived from PGS, as well as the proportion of at-risk individuals identified by PGS vs rCNV groups for each disorder. Point estimates with error bars represent absolute risks and their corresponding 95% CI for low, middle, and high rCNV groups (Figure 1). In each panel, the density plot represents an artificial PGS distribution that was generated by sampling 100 000 quantiles based on the mean (SD) observed in the random subcohort for each disorder. Expected absolute risk was calculated for each quantile, and the proportion of individuals with PGS-derived risk exceeding rCNV-associated risk was determined from the upper tail of the distribution (Methods and eMethods in Supplement 1). The upper x-axis shows absolute risk values for both PGS and rCNV groups, whereas the lower x-axis indicates the proportion of individuals in the PGS distribution with equal or greater absolute risk than the corresponding numbers indicated on the upper x-axis. invLOEUF indicates inverted loss of function observed/expected upper fraction. ^a^For each rCNV group, footnote indicates the corresponding proportion of individuals in the PGS distribution with equal or greater absolute risk.

We next examined for each disorder the expected proportion of individuals in our reference population (ie, Denmark) with PGS-based risk equivalent to or higher than that estimated for the 3 rCNV-carrier groups; representing 1.4%, 0.55%, and 0.35% of the population for low-, moderate-, and high-impact rCNVs, respectively (Figure 5 and eTables 15 and 16 in Supplement 1). For ASD, this proportion was smaller (0.06%, <0.02%, <1 × 10^−7^%, respectively) than the proportion of individuals carrying rCNVs at each group level (as detailed previously), whereas the opposite was true for the other 3 disorders, with the corresponding proportions ranging between 19.9% and 42.4%, 3.6% and 37.3%, and 0.55% and 9.6%, respectively. We conducted similar comparisons for 6 commonly studied rCNVs at 15q11.2, 16p13.11, and 22q11.2 (eFigures 5 and 6 and eTable 17 in Supplement 1) and found that for all except the most penetrant rCNVs (eg, 22q11.2 deletion and SSD), PGSs identified more individuals with comparable genetic risk, with the exception of ASD, where PGS-based risk rarely reached that conferred by rCNVs.

### Polygenic Profiles and Etiological Heterogeneity Associations With rCNV Carriers

Lastly, we investigated the polygenic background of rCNV carriers to explore 3 hypotheses. First, we asked if among cases the primary disorder PGS (ie, ADHD PGS for ADHD cases) was lower in rCNV carriers than noncarriers. This was true for ASD and ADHD, with ADHD rCNV carriers also showing incremental decrease in ADHD-PGS across rCNV groups of increasing impact, but in no instance were the differences significant (eFigure 7 and eTable 19 in Supplement 1 and eTable 18 in Supplement 2). Next, we asked if among rCNV carriers (combined or grouped by impact), the PGS for the observed outcome was higher than for any of the other outcomes. This was true in 7 of the 16 tested rCNV-outcome combinations, but in no instance was the difference significant (eFigures 7 and 8 and eTable 19 in Supplement 1 and eTable 18 in Supplement 2). Finally, we asked if rCNV carriers had a qualitatively different PGS profile from noncarriers (as suggested by a previous study^31^). Comparing 13 PGSs among the 16 rCNV-outcome combinations, we again found no significant evidence for differences (eTable 20 in Supplement 1 and eTables 21 and 22 in Supplement 2).

## Discussion

Using the population-representative iPSYCH2015 case-cohort,^38^ we estimated absolute risks associated with rCNVs and PGSs across ASD, ADHD, SSD, and MDD, independently and jointly. To our knowledge, this was the first study to do so in ASD and the largest for ADHD and MDD. We also compared the proportion of individuals reaching comparable absolute risk via PGSs vs rCNVs across the 4 disorders and explored whether polygenic profiles of rCNV carriers suggest distinct etiological subgroups.

Aligned with our previous research,^8^ higher-impact rCNVs (including from 9 loci not assessed in our previous study) were associated with elevated absolute risk of ASD, ADHD, and SSD but not MDD.^8^ As expected, higher PGSs were associated with increased absolute risk for all 4 disorders.^14,32,50^ Among those, ASD-PGS was least predictive, as also found in previous studies.^16,17,18,51^

Our findings highlight the complementary roles of rCNVs and PGSs in risk prediction in psychiatry. For example, among carriers of medium- and high-impact rCNVs, the difference in absolute risk between the high and low PGS groups was generally larger than among noncarriers, suggesting a particular translational potential of PGSs for predicting risk among rCNV carriers for ASD, ADHD, and SSD, although this was not confirmed by statistical tests for interactions. For SSD, the low PGS group showed minimal increases in risk over noncarriers across rCNV carrier groups. These observations align with evidence from other fields, where PGSs can refine risk stratification in carriers of high-impact variants, including in some cancers and familial hypercholesterolemia.^24,52^ Moreover, since many rCNVs have a pleiotropic effect across several psychiatric disorders, integrating rCNV and PGS risk assessment could improve the nosological specificity of risk prediction and stratification in clinical and research contexts, and continued integration of genetic data with neurodevelopmental, cognitive, and environmental measures will likely further enhance the accuracy and utility of such efforts.

When considered separately, PGS in most instances identified more individuals at comparable or higher risk than when considering rCNV carrier status alone—mirroring previous studies of PGS and rare pathogenic gene variants in other complex traits, such as cardiometabolic disease or cancer,^20^ although it should be highlighted that differences in the estimated fraction of individuals in the population at comparable PGS/rCNV–based risk could not be formally tested in a statistical framework. In this comparison ASD was again an outlier, with rCNV carriage outcompeting PGS in risk prediction at every level. This in part reflects current limitations of ASD-PGS, including reduced power from having to rely on GWAS that do not include the iPSYCH2015 sample.^16^ With better-powered GWAS and more robust PGS development, it is likely that the trends for ASD will match those of the other disorders, and with improved methods to detect and study CNVs and rare sequence variants, the combined predictive power across all variant classes will also improve.

In this work, we focused on describing the joint and relative association of rCNVs and PGSs with psychiatric disorders in population-representative data and how combining both risk factors improves risk stratification. Resolving the etiological model—eg, if rCNVs and PGSs act additively in liability threshold model, if rCNVs create a general vulnerability that amplifies disorder-specific PGS effects, or if rCNV carriers may have a wholly different syndrome—that underlies such joint contributions is difficult, as is interpreting interaction tests, which can depend on the scale of measurement.^53^ If rCNVs and PGSs acted additively in a liability threshold model, the nonlinear thresholding of liability predicts both a negative interaction between rCNVs and PGSs in a logistic regression,^53^ and rCNV carriers would, on average, have a lower PGS. Previous literature on this issue has been mixed, although a handful of studies of ADHD^30^ and schizophrenia^31,32,34^ has reported lower PGSs in rCNV carriers and/or a negative interaction. We found only limited evidence for negative interactions and reduced PGS burden, and although we observed a trend for negative interaction coefficient across all tests, only the interaction for ADHD-PGS and 16p13.11 duplication was significant at a single-test level. None of the rCNV carrier groups had significantly reduced PGS burden for any of the 4 disorders. These discrepancies may reflect differences in study design and/or low power. Clinical case-control studies, including that of Bergen et al,^32^ often involve severely affected cases with long-term illness and healthy controls screened for family history of mental illness. This design tends to overestimate effect sizes through enriching both ends of the genetic risk distribution. For example, our previous population-based studies^6,7,8^ found lower SSD risk estimates for several rCNVs than reported in case-control studies.^5,54^

We also found no support for earlier reported extreme heterogeneity in the genetic background of rCNV carriers^31^ in our comparison across other psychiatric, cognitive, and selected somatic traits.

### Limitations

This study has some limitations. The rarity of rCNVs limits the power to detect their interactions with PGSs, and although we mitigate this challenge by grouping rCNVs based on summed invLOEUF scores of affected genes, this may mask any locus-specific effects. Also, although we deem this to be the most objective available measure for grouping rCNVs by expected pathogenicity, given the lack of external risk estimates for many variants, the consideration of a few grouping approaches before selecting invLOEUF may have led to modest overestimation of rCNV group effects. Our reliance on hospital-based registry diagnoses misses cases diagnosed outside the hospital system, particularly for MDD. PGS performance, especially for ASD, was constrained by the exclusion of iPSYCH samples from GWAS discovery sets, and the relatively young age of the cohort limits our ability to assess lifetime risk, especially for SSD and MDD. Lastly, our findings are limited to individuals of European ancestry.

## Conclusions

Findings of this genetic association study highlight the complementary value of rCNVs and PGS for risk assessment in psychiatric disorders. Although some rCNVs remain important in clinical genetics, given their high penetrance and interpretability for some disorders, PGSs in most instances were able to identify a larger proportion of the population with equivalent or greater risk in ADHD, SSD, and MDD, which parallels recent results for PGSs in somatic domains. Future research should aim to refine PGSs through larger and more diverse GWAS, incorporate rCNV analysis into routine screening in high-risk populations, and explore multimodal approaches combining genetic, neurobiological, and environmental data.

## Notes

Supplement 1.
eMethods.
**eFigure 1.** Associated Odds Ratios and Confidence Intervals Resulted From the Sensitivity Analysis for Grouping rCNVs Across 6 Different Approaches **eFigure 2.** Comparison of rCNV Group-Associated Absolute Risk Between SSD and SSD Without Comorbid Childhood-Onset Disorders in iPSYCH2015 **eFigure 3.** Comparison of PGS Group-Associated Absolute Risk Between SSD and SSD Without Comorbid Childhood-Onset Disorders in iPSCYH2015 **eFigure 4.** Comparison of Absolute Risk Associated With Joint rCNV and PGS Groups for SSD and SSD Without Comorbid Childhood-Onset Disorders in the iPSYCH2015 **eFigure 5.** Comparison of Absolute Risk Estimates and Proportion of At-Risk Individuals Attributed to 6 Common rCNVs and Disorder-Specific PGS for ASD, ADHD, SSD, and MDD in the iPSYCH2015 Random Cohort **eFigure 6.** Calibration Analysis for Disease-Specific PGSs in iPSYCH2015 **eFigure 7.** Comparison of Mean PGS Between rCNV Carriers and Noncarriers Across Cases and Controls **eFigure 8.** Comparison of Disease-Specific PGS Among rCNV Carriers and Noncarriers Across Different Diagnostic Groups **eTable 1.** rCNV Loci Used in the Study and Their Corresponding invLOUEF Score Group **eTable 6.** Number of rCNV Carriers Within invLOEUF Groups and Collectively Across Cohort and Cases in iPSYCH2015 Case-Cohort (Unrelated European Sample) **eTable 7.** Associated Effect Sizes and Standard Errors of rCNV-invLOEUF Groups and PGS on ASD, ADHD, SSD, and MDD Derived From Fitted GLMs **eTable 9.** Model Fitting Results for ASD, ADHD, SSD, and MDD as a Function of PGS and rCNV Status (as invLOEUF Groups and Aggregated) **eTable 10.** Model Fitting Results for Schizophrenia (SCZ) and SSD Without Comorbid Childhood-Onset Disorders as a Function of PGS and rCNV Status (Using rCNVs as invLOEUF Groups and Aggregated) **eTable 13.** CNV×PRS Interaction on ASD, ADHD, and SSD for rCNV-invLOEUF Groups, Aggregated rCNVs, and 9 Common Individual rCNVs **eTable 14.** Number of Carriers of the Common rCNVs in iPSYCH2015 Across Cohort and Case Samples (Unrelated European Sample) **eTable 15.** Comparison of Odds Ratios (OR) and Risk Ratios (RR) Attributed to Disease-Specific PGS Across ASD, ADHD, SSD, and MDD **eTable 16.** Proportion of Individuals Whose PGS-Derived Absolute Risk Exceeds the Risk Associated With rCNV Groups Across ASD, ADHD, SSD, and MDD **eTable 17.** Proportion of Individuals Whose PGS-Derived Absolute Risk Exceeds the Risk Associated With 6 Common rCNVs Across ASD, ADHD, SSD, and MDD **eTable 19.** LRT Results for Comparison of Disorder-Specific PGSs Between rCNV Carriers and Noncarriers Across ASD, ADHD, SSD, and MDD **eTable 20.** Results From LRTs Comparing Overall Psychiatric, Behavioral, and Somatic PGS Profiles Between rCNV Carriers and Noncarriers
eReferences.

Supplement 2. **eTable 2.** Grouping rCNVs Into Different Risk Groups Across 6 Approaches for Sensitivity Analysis **eTable 3.** Association Analyses of rCNV Groups Across Different Grouping Approaches for ASD, ADHD, SSD, and MDD **eTable 4.** Associated ORs and 95% CIs With 34 rCNVs for ASD, ADHD, SSD, and MDD in iPSYCH2015 Case-Cohort Used for Sensitivity Analysis **eTable 5.** Associated ORs and 95% CIs With 34 rCNVs for ASD, ADHD, SSD, and MDD Reported in the Literature Used for Sensitivity Analysis **eTable 8.** Number of Individuals Across PGS Strata and rCNV Groups in Case-Control Analyses for Each Diagnostic Category **eTable 11.** Model Fitting Results for ASD, ADHD, SSD, and MDD as a Function of PGS and rCNV Status in Carriers of 9 Common rCNVs in iPSYCH2015 **eTable 12.** Model Fitting Results for SSD Without Comorbid Childhood-Onset Disorders as a Function of PGS and rCNV Status in Carriers of 9 Common rCNVs in iPSYCH2015 **eTable 18.** Average PGS for Carriers and Noncarriers Across ASD, ADHD, SSD, and MDD by Affection Status **eTable 21.** Summary of Fitted GLMs Assessing Differences in PGS Profiles Related to Psychiatric, Behavioral, and Somatic Traits Between rCNV Carriers and Noncarriers **eTable 22.** Pairwise Correlations of Polygenic Scores (PGSs) Across 13 Traits in the Study Sample

Supplement 3. **Nonauthor Collaborators.** LINC Consortium.

Supplement 4.
Data Sharing Statement.
