# Supplementary material for: Recurrent Copy Number Variants and Psychiatric Outcomes in the Context of Polygenic Scores
Source: JAMA Psychiatry. 2026 May 27;83(8):827–36. doi: 10.1001/jamapsychiatry.2026.1064 (PMC13217261; doi:10.1001/jamapsychiatry.2026.1064)
Supplement: Supplement 1. — eMethods. eFigure 1. Associated Odds Ratios and Confidence Intervals Resulted From the Sensitivity Analysis for Grouping rCNVs Across 6 Different Approaches eFigure 2. Comparison of rCNV Group-Associated Absolute Risk Between SSD and SSD Without Comorbid Childhood-Onset Disorders in iPSYCH2015 eFigure 3. Comparison of PGS Group-Associated Absolute Risk Between SSD and SSD Without Comorbid Childhood-Onset Disorders in iPSCYH2015 eFigure 4. Comparison of Absolute Risk Associated With Joint rCNV and PGS Groups for SSD and SSD Without Comorbid Childhood-Onset Disorders in the iPSYCH2015 eFigure 5. Comparison of Absolute Risk Estimates and Proportion of At-Risk Individuals Attributed to 6 Common rCNVs and Disorder-Specific PGS for ASD, ADHD, SSD, and MDD in the iPSYCH2015 Random Cohort eFigure 6. Calibration Analysis for Disease-Specific PGSs in iPSYCH2015 eFigure 7. Comparison of Mean PGS Between rCNV Carriers and Noncarriers Across Cases and Controls eFigure 8. Comparison of Disease-Specific PGS Among rCNV Carriers and Noncarriers Across Different Diagnostic Groups eTable 1. rCNV Loci Used in the Study and Their Corresponding invLOUEF Score Group eTable 6. Number of rCNV Carriers Within invLOEUF Groups and Collectively Across Cohort and Cases in iPSYCH2015 Case-Cohort (Unrelated European Sample) eTable 7. Associated Effect Sizes and Standard Errors of rCNV-invLOEUF Groups and PGS on ASD, ADHD, SSD, and MDD Derived From Fitted GLMs eTable 9. Model Fitting Results for ASD, ADHD, SSD, and MDD as a Function of PGS and rCNV Status (as invLOEUF Groups and Aggregated) eTable 10. Model Fitting Results for Schizophrenia (SCZ) and SSD Without Comorbid Childhood-Onset Disorders as a Function of PGS and rCNV Status (Using rCNVs as invLOEUF Groups and Aggregated) eTable 13. CNV×PRS Interaction on ASD, ADHD, and SSD for rCNV-invLOEUF Groups, Aggregated rCNVs, and 9 Common Individual rCNVs eTable 14. Number of Carriers of the Common rCNVs in iPSYCH2015 Across Cohort and Case Samples [file jamapsychiatry-e261064-s001.pdf]

Supplementary Online Content

Vaez M, Montalbano S, Waples R, et al; LINC Consortium. Recurrent copy number variants and psychiatric outcomes in the context of polygenic scores. *JAMA Psychiatry*. Published online May 27, 2026. doi:10.1001/jamapsychiatry.2026.1064

eMethods.

eFigure 1. Associated Odds Ratios and Confidence Intervals Resulted From the Sensitivity Analysis for Grouping rCNVs Across 6 Different Approaches

eFigure 2. Comparison of rCNV Group-Associated Absolute Risk Between SSD and SSD Without Comorbid Childhood-Onset Disorders in iPSYCH2015

eFigure 3. Comparison of PGS Group-Associated Absolute Risk Between SSD and SSD Without Comorbid Childhood-Onset Disorders in iPSCYH2015

eFigure 4. Comparison of Absolute Risk Associated With Joint rCNV and PGS Groups for SSD and SSD Without Comorbid Childhood-Onset Disorders in the iPSYCH2015

eFigure 5. Comparison of Absolute Risk Estimates and Proportion of At-Risk Individuals Attributed to 6 Common rCNVs and Disorder-Specific PGS for ASD, ADHD, SSD, and MDD in the iPSYCH2015 Random Cohort

eFigure 6. Calibration Analysis for Disease-Specific PGSs in iPSYCH2015

eFigure 7. Comparison of Mean PGS Between rCNV Carriers and Noncarriers Across Cases and Controls

eFigure 8. Comparison of Disease-Specific PGS Among rCNV Carriers and Noncarriers Across Different Diagnostic Groups

eTable 1. rCNV Loci Used in the Study and Their Corresponding invLOUEF Score Group

eTable 6. Number of rCNV Carriers Within invLOEUF Groups and Collectively Across Cohort and Cases in iPSYCH2015 Case-Cohort (Unrelated European Sample)

eTable 7. Associated Effect Sizes and Standard Errors of rCNV-invLOEUF Groups and PGS on ASD, ADHD, SSD, and MDD Derived From Fitted GLMs

eTable 9. Model Fitting Results for ASD, ADHD, SSD, and MDD as a Function of PGS and rCNV Status (as invLOEUF Groups and Aggregated)

© 2026 Vaez M et al. *JAMA Psychiatry*.

eTable 10. Model Fitting Results for Schizophrenia (SCZ) and SSD Without Comorbid Childhood-Onset Disorders as a Function of PGS and rCNV Status (Using rCNVs as invLOEUF Groups and Aggregated)

eTable 13. CNV×PRS Interaction on ASD, ADHD, and SSD for rCNV-invLOEUF Groups, Aggregated rCNVs, and 9 Common Individual rCNVs

eTable 14. Number of Carriers of the Common rCNVs in iPSYCH2015 Across Cohort and Case Samples (Unrelated European Sample)

eTable 15. Comparison of Odds Ratios (OR) and Risk Ratios (RR) Attributed to Disease-Specific PGS Across ASD, ADHD, SSD, and MDD

eTable 16. Proportion of Individuals Whose PGS-Derived Absolute Risk Exceeds the Risk Associated With rCNV Groups Across ASD, ADHD, SSD, and MDD

eTable 17. Proportion of Individuals Whose PGS-Derived Absolute Risk Exceeds the Risk Associated With 6 Common rCNVs Across ASD, ADHD, SSD, and MDD

eTable 19. LRT Results for Comparison of Disorder-Specific PGSs Between rCNV Carriers and Noncarriers Across ASD, ADHD, SSD, and MDD

eTable 20. Results From LRTs Comparing Overall Psychiatric, Behavioral, and Somatic PGS Profiles Between rCNV Carriers and Noncarriers

eReferences.

This supplementary material has been provided by the authors to give readers additional information about their work.

eMethods: Detailed methods description

Merging genotyped samples on two arrays:

Samples genotyped on both arrays were first phased and imputed using BEAGLE5.4 separately, with HRCv1.1[1] as the reference. Afterwards, SNPs from two separately imputed datasets were selected if they passed the following criteria: 1) Genotyped at least on one of the two arrays; 2) Minimum imputation quality above 0.99 within both datasets, and 3) Maximum of 1.5% different minor allele frequency (MAF) between the two datasets. The selected SNPs from both datasets were ultimately phased and imputed again using BEAGLE5.4, aligning with HRCv1.1,[1], resulting in a total of 6,518,119 SNPs with a minimum MAF>1% after performing quality control.

Identifying unrelated individuals of European ancestry from the genotypic data:

© 2026 Vaez M et al. *JAMA Psychiatry*.

Ancestry information is not available for the iPSYCH individuals.[2] Thus, to identify subjects with a homogenous origin, the 1000 Genomes phase 3 dataset[3] of variants was first downloaded in VCF format, and multiple steps of QC were performed on the sets of variants afterward. In the QC steps, those SNPs with a minor allele frequency lower than 5%, Hardy–Weinberg p values $<10^{-6}$, pairwise correlation (r2) >0.1 within a 1 kb region were excluded. Additionally, SNPs that did not belong to either the Infinium psych chip v1.0 or the Illumina global screening array v2.0, SNPs in regions with extended linkage disequilibrium, as well as insertions or deletions, were removed from the data. Afterward, the QC-ed data were merged with both iPSYCH2012[4] and iPSYCH2015i datasets, and iPSYCH samples were furthermore projected into the principal components computed for 1000 Genomes subjects. To distinguish the samples of European and Danish ancestry, 47,586 individuals within the iPSYCH2012 sample[4] with both Danish parents and grandparents were identified using Danish civil registers[5], for which the first 10 principal components were then obtained. For each sample, the Mahalanobis distance was computed concerning the first 10 PCs corresponding to the previously identified 47,586 subjects. A sample was retained if the distance had a probability of more than 5.73×10^{-7} under a chi-square distribution. After performing genetic ancestry QC, 73,052 samples in iPSYCH2012 and 47,217 samples in iPSYCH2015i were flagged as local genetic ancestry inliers. Subsequently, kinship coefficients of the sample were estimated utilizing KING[6], and individuals beyond the thirddegree relatedness were pruned.[7] Ultimately, 107,716 unrelated individuals in iPSYCH2015 were deemed to have European-Danish ancestry, which we used for all analyses in this study.

CNV Grouping strategies and sensitivity analysis:

We performed a series of sensitivity analyses to assess whether grouping individual rCNVs based on various methods into low to high-risk groups would reflect a similar pattern of distinct penetrance across the four diagnoses, namely, ASD, ADHD, SSD, and MDD. We explored and compared 6 grouping methods as follows:

1. Odds ratios in iPSYCH2015: Here, we grouped rCNVs based on their odds ratios (ORs) for each diagnosis previously estimated in iPSYCH2015.[8] For ASD, ADHD, and SSD, rCNVs were divided into three levels using the following cutoffs; OR≤1, 1<OR<2, and OR≥2, where rCNVs were labeled as "no evidence of pathogenicity"," low risk"," medium risk" and "high risk", and coded as "0", "1", "2" or "3", respectively. However, for MDD, since there was no indication of significant rCNV association,[8] rCNVs were split into two groups; namely "low risk" and "high risk", with OR of 1 set as the cut-off and coded as "1" and "2", respectively.
2. Odds ratios in the literature:[9-14] rCNVs were divided using the external ORs, wherever available. rCNVs with OR≥2 (coded as 2) for the corresponding disorder were grouped as high risk, otherwise labeled as low risk for those with OR<2 or a missing risk estimate (coded as 1).
3. ClinGen haploinsufficiency/ triplosensitivity index:[15] We defined an rCNV as high risk if it was identified by the ClinGen website as a rCNV with some evidence for haploinsufficiency/ triplosensitivity, else as a low-risk rCNV across the 4 outcomes (i.e., low risk and high risk were labeled with scores 1 and 2, respectively).
4. invLOEUF score:[16] rCNVs were split into three groups with regard to their inverse LOEUF scores (invLOEUF) as low invLOEUF (<10), medium invLOEUF (10-25), and high invLOEUF (>25) corresponding to the arbitrary scores "1","2", and "3", respectively.
5. Composite score (CS): Composite scores for each rCNV were generated by summing up their given scores within each of the three latter approaches, namely, external ORs, [9-14] ClinGen,[15] as well as invlOEUF score, resulting in CS scores ranging from 3 to 7, which were then converted to 1,2,3 corresponding to low, medium, and high-risk groups ultimately.

6. Any CNV vs. no CNV: Lastly, we collapsed all the rCNV carriers at 27 loci in one aggregated group (i.e, carriers of any rCNV vs non-carriers).

We then calculated the risk estimates associated with the rCNV group for predicting each diagnosis using generalized linear models (GLMs) across the 6 grouping approaches. In each model, rCNV status was used as the categorical explanatory variable accounting for age, sex, and sample origin (i.e., iPSYCH2012 or iPSYCH2015i). rCNV status was defined as being an rCNV carrier belonging to the CNV group, with the reference set as "noncarriers". Only cases diagnosed with the respective outcome or controls from the random cohort entered the

© 2026 Vaez M et al. *JAMA Psychiatry*.

relevant analysis. To be consistent in grouping rCNVs across different grouping methods, we restricted the sensitivity analyses to 34 rCNVs at 18 loci that had corresponding risk estimates associated with the four diagnoses reported in our previous study within iPSYCH2015.[8]

Polygenic scores:

To compute PGSs, we used the latest external GWAS summary statistics [17-29] for all the phenotypes in this study. For ASD, ADHD, SCZ, and MDD, we obtained GWASs from the Psychiatric Genomics Consortium (PGC)[30] after excluding iPSYCH individuals from the discovery sample.[17-20] The number of SNPs used for PGS generation was 1,041,920 after intersecting SNPs in iPSYCH2015 datasets with SNPs in HapMap3[31] as the reference. The p-value threshold for SNPs in each GWAS was set at 0.9. SNP effects reported in each GWAS were rescaled using SBayesR[32], based on Bayesian multiple regression with the scaling factor (γ) and the number of mixture components (π) set to the default settings (--pi 0.95,0.02,0.02,0.02,0.02,0.01; --gamma 0,0.01,0.1,1). After rescaling the SNP's effect, PGSs were generated by applying the *--score* module in *PLINKv2.00a2.3LM* to the *SBayesR's* output files.

Absolute Risk of ASD, ADHD, SSD, and MDD Associated with rCNV and PGS Groups in iPSYCH2015 Case-Cohort

The rCNV-associated absolute risk for each diagnosis was derived using the *survfit* function from the R survival package[33], with age at first diagnosis or censoring age, depending on the event proximity, as the outcome and rCNV carrier status as the explanatory variable in each model. For each disorder-specific analysis, we included all individuals diagnosed with the index disorder as cases, together with all individuals from the random subcohort. Individuals overlapping between the case sample and the random subcohort were classified according to their status for the index disorder: those with the disorder were treated as cases, whereas those without the index diagnosis were treated as controls, consistent with other members of the random subcohort. Censorship was implemented such that if there was a death, emigration, or no diagnosis of any iPSYCH index disorder by the end of the follow-up period (December 31st, 2015), or if the participant was lost to follow-up. rCNV status was defined as a categorical variable with "no rCNV" as the reference in each model. Similar models were also used when investigating absolute risk associated with PGS groups as the explanatory variable, where the lowest PGS group (i.e., PGS<20%) was the reference for this categorical variable. The definition of variables was furthermore the same when using both rCNV and PGS groups as independent variables for estimating absolute risks. All models accounted for the inverse probability of sampling (IPS) weights, following the weighting method developed by Barlow et al[34]. Due to legal restrictions on sharing individual-level data, we smoothened absolute risk curves by applying the *cobs* function from the cobs package[35] in R to the derived survival data from each fitted model.

To account for population stratification effects, all PGS calculations were linearly regressed against the first 20 PCs of unrelated individuals of European ancestry, and the residuals of fitted models were subsequently preserved for analysis.

Assessment of polygenic score calibration:

Calibration of the PGS was assessed by comparing observed and model-predicted outcome probabilities across the distribution of each outcome-specific PGS. For each outcome (ASD, ADHD, SSD, and MDD), a global logistic regression model was first fitted with the corresponding PGS as a predictor while accounting for 20 prinicple components (PCs), yielding predicted probabilities for all individuals. Second, individuals were then grouped into 5% cumulative intervals based on the distribution of the relevant PGS. Within each PGS interval, an intercept-only logistic regression model was fitted to estimate the observed outcome probability for that bin. Expected probabilities were obtained from the global logistic regression model by evaluating the predicted probability at the mean PGS value within each bin. Observed and expected probabilities were compared across PGS bins to assess agreement between predicted and empirical outcome frequencies. Calibration was evaluated descriptively using graphical comparison of observed and predicted probabilities, with uncertainty in observed probabilities reflected by 95% CI (see eFigure 8).

© 2026 Vaez M et al. *JAMA Psychiatry*.

Generating individual-level Absolute Risk for ASD, ADHD, SDD, and MDD using PGSs:
We sought to estimate the proportion of the population with as much or more PGS-derived risk as rCNVs. First, we estimated the population absolute risk (P_0) and absolute risk conferred by each rCNV (AR_{CNV}) using weighted survival models as described previously. Second, we estimated the effect of each individual PGS (β_{pgs}) on their target disorder using logistic regression while adjusting for age, sex, and genotyping array. Third, we constructed an artificial representation of the PGS distribution in the Danish population by direct sampling, under the assumption that the PGS is well calibrated in the target population. We estimated the mean (μ_{pgs}) and standard deviation (σ_{pgs}) of the PGS distribution in the random subcohort of iPSYCH, and then used internal functions in R to sample 100,000 representative quantiles using the command *qnorm(seq(*μ_{pgs}*,* σ_{pgs}*, by=100000))*. This approach is privacy protecting as Danish law prohibits publishing individual quantiles and associated cumulative distribution function probabilities from empirical distributions. Fourth, for each representative PGS quantile (pgs_Q), we estimated the expected absolute risk using P_0 and β_{pgs} as follows:

$$RR_{pgs\_Q} = \frac{exp(\ pgs_Q \times \beta pgs)}{\left(1 - P0 + (\ P0 \times pgs_Q \times \beta pgs)\right)}$$

$$AR_{pgs\_Q} = P_0 \times RR_Q$$

Finally, we selected the minimum sampled pgs quantile for which the $AR_{pgs\_quantile}$ was greater than the AR_{CNV} and report the proportion of individuals as the upper tail cumulative probability associated with that quantile (i.e., 1 - pnorm($pgs_{quantile}$, mean=μ_{pgs}, sd=σ_{pgs})).

The same analyses were repeated using the lower and upper confidence interval bounds of β_{pgs} to provide a crude estimate of sampling variance for the estimated proportions. Additionally, we conducted similar comparison analyses using 6 individual rCNVs (i.e, rCNVs at 15q11.2, 16p13.11, and 22q11.2) instead of rCNV groups.

Fitting generalised linear models (GLMs):

We constructed a series of logistic regression models by GLM to investigate the effect of rCNV and PGS on the risk of each outcome, separately. All models were adjusted for age, sex, and sample origin (i.e., iPSYCH2012 or iPSYCH2015i) of subjects, where the model outcome was the status for the target diagnosis coded as a binary variable. Sequentially, we introduced rCNV and PGS as predictors into each model, assessing them individually, in combination (i.e., rCNV + PGS), as well as in the interactive form of rCNV×PGS. In each step, a likelihood ratio test was employed to compare the fitted model with the nested model. For each diagnosis, rCNVs were analyzed individually, in the form of invLOEUF-groups and aggregate (i.e., any rCNV), as well as 9 common rCNVs individually as a categorical variable, where the carrier status was labeled with regard to the reference level as having no rCNV. Furthermore, to test the effect of levels of invLOEUF groups on each outcome, we fitted similar GLMs, where rCNV groups (i.e., invLOEUF groups) were included as a numeric covariate after converting the invLOEUF groups' levels to 0, 1, 2, and 3, representing "having no rCNV", rCNVs with "invLOEUF<10", "10<invLOEUF<25", and "invLOEUF>25", respectively. When assessing the PGS effect using PGS as a linear variable as well as the categorical variable with three strata (i.e., less than 20th percentile, between 20th and 80th percentile, and over 80th percentile). To adjust for the population stratification, all PGS scores were regressed on 20 principal components from unrelated European individuals, and the residuals were retained for further analysis. We derived Nagelkerke's R-squared for each model by subtracting the R-squared of the fitted model from the R-squared of the null model, utilizing the *PseudoR2* function from the DescTools package[36].

Subsequently, to compute the standard errors (SE) of Nagelkerke's R-squared obtained from each fitted model, we first bootstrapped the sample 1000 times, followed by fitting the model each time, and deriving their corresponding Nagelkerke's R-squared. We then estimated the SE of R-squared by deriving the SD of the R-squared vector corresponding to the bootstrapped models divided by the square root of 1000. We used Nagelkerke's R^2 primarily to provide an approximate measure of variance explained by each predictor for illustrative purposes, complementing

© 2026 Vaez M et al. *JAMA Psychiatry*.

formal statistical comparisons conducted via likelihood ratio tests (LRTs), which remain the rigorous basis for model comparisons.[37-38]

To estimate the joint effects of rCNV groups and PGS under additive and multiplicative assumptions, we fitted GLMs using the same outcome definition and covariates as described above. The reference groups were individuals without rCNVs and those with PGS percentiles between 20 and 80. Joint odds ratios (ORs) for high-impact rCNVs (invLOEUF > 25) and high PGS (PGS > 80th percentile) were derived under the additive assumption as:

$OR_{invLOEUF<25} + OR_{PGS>80\%} - 1$ and under the

multiplicative assumption as:

$OR_{invLOEUF>25} \times OR_{PGS>80\%}$

Finally, the observed interaction effect between the two genetic predictors was estimated from GLMs including an interaction term, by summing the individual ORs for high-impact rCNVs and high PGS together with the OR attributed to their interaction.

Comparison of polygenic profile between rCNV carriers and non-carriers:

To examine whether PGS for psychiatric disorders differs between rCNV carriers and non-carriers, we fitted several models for each outcome separately. For the aggregated group of rCNV carriers (i.e., any rCNV carriers vs. noncarriers), we implemented a nested GLM that included age, sex, and the first 20 PCs as covariates, with rCNV status as the binary outcome and non-carriers as the reference group. The full model additionally incorporated the PGS corresponding to the psychiatric outcome as an independent predictor. The model differences were tested employing LRTs.

We then conducted similar analyses among carriers categorized by invLOEUF groups, using linear regression models. Here, rCNV status was treated as a numeric outcome by assigning values to rCNV levels as follows: no CNV = 0, low invLOEUF = 1, middle invLOEUF = 2, and high invLOEUF = 3.

To examine differences in the combined polygenic architecture across 13 phenotypes between aggregated rCNV carriers and non-carriers, we fitted a series of GLMs, using the same covariate structure and model comparison approach as in prior analyses. To do so, we generated PGSs for BPD, five behavioural (risk-taking behavior, neuroticism, alcoholism, intelligence, and educational attainment, as well as somatic traits (diabetes, coronary artery disease, and body mass index (BMI)). Then, we sequentially added groups of PGSs to the nested model: first, five PGSs for psychiatric disorders (i.e., ADHD, ASD, MDD, SCZ, and BPD); next, five PGSs for behavioral traits; and finally, three PGSs for somatic traits.

To extend these analyses to rCNV-invLOEUF groups, we used multinomial regression models from the nnet package[39] in R, constructing a similar base model as in the previous section. This time, all 13 PGSs were simultaneously added to the full model. LRTs were applied in all steps to test the model fit, and each analysis was conducted separately within the case-control sample for ASD, ADHD, SSD, and MDD

Supplementary Figures

eFigure 1: *Associated Odds ratios and confidence intervals resulted from the sensitivity analysis for grouping rCNVs across 6 different approaches.*

© 2026 Vaez M et al. *JAMA Psychiatry*.

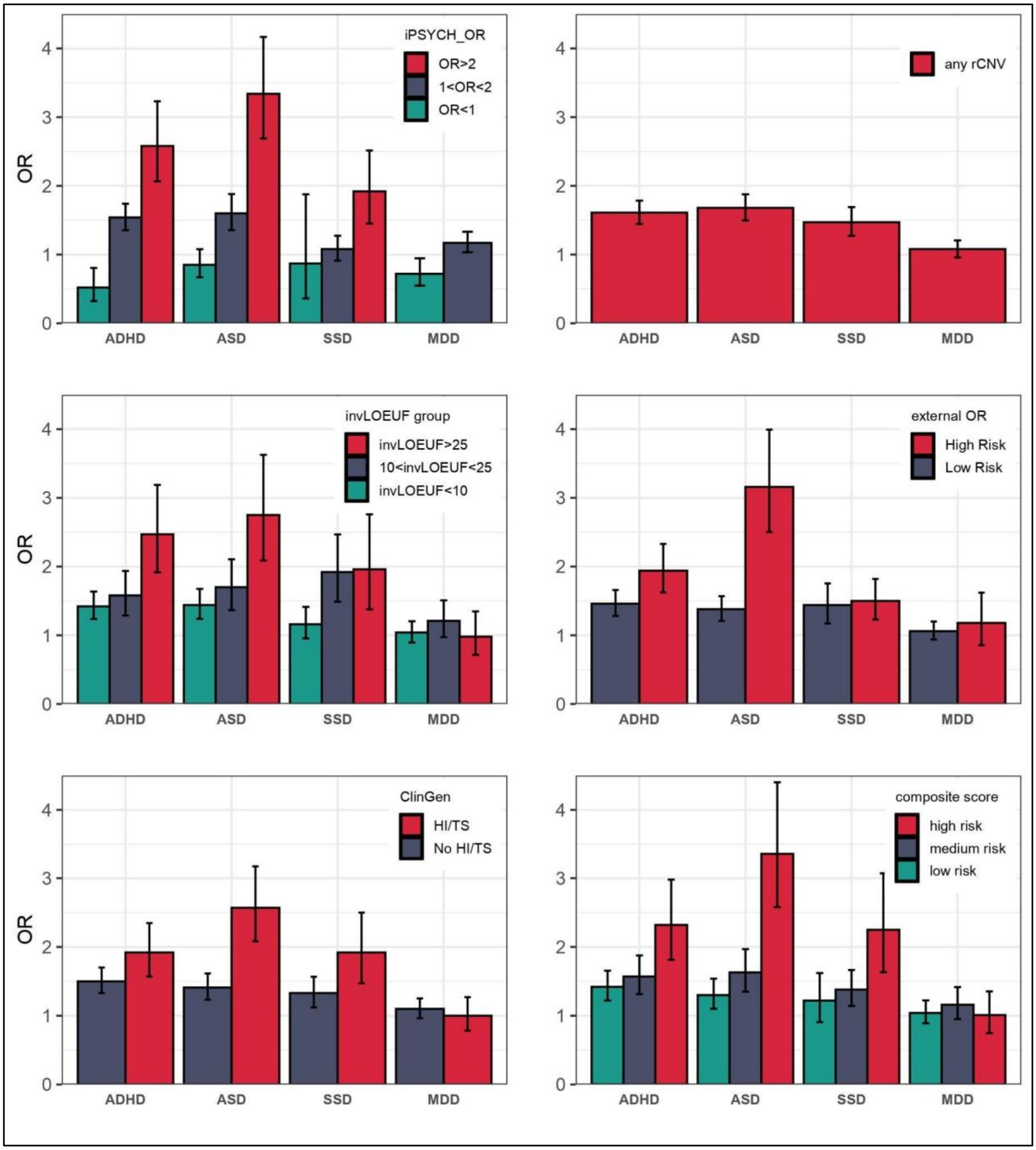

We conducted six different analyses to identify the most objective and least biased strategy for grouping rCNVs, while preserving variation in penetrance across disorders and maintaining sufficient power to study the joint contribution of rCNVs and PGS. rCNV group-associated odds ratios (ORs) and confidence intervals (CI95%) were derived from generalized linear models (GLMs) across six different grouping approaches for each diagnosis, separately. GLM results from rCNV grouping using rCNV-associated ORs in iPSYCH2015 for each disorder, any rCNV vs. no rCNV approach, rCNVs invLOEUF scores, rCNV-associated external ORs for each disorder, indication of haploinsufficiency or triplosensitivity of rCNVs in ClinGen[15], and generated composite scores derived from the three latter approaches (i.e., invLOEUF score threshold, external ORs, and ClinGen) are shown on the upper right, upper left, middle right, middle left, lower left, and lower right, respectively (see eMethod, eTable 1-6). The red, dark gray, and dark green colors of the bars represent different rCNV groups within each approach. Error bars indicate 95% CI corresponding to ORs. ASD; autism spectrum disorder, ADHD; attention-deficit hyperactivity disorder, SSD; schizophrenia spectrum disorder, MDD; major depressive disorder. Given that all evaluated strategies yielded risk stratification patterns broadly consistent with our initial

© 2026 Vaez M et al. *JAMA Psychiatry*.

approach—based on rCNV-associated odds ratios estimated in the iPSYCH dataset—we ultimately grouped rCNVs according to the summed invLOEUF scores of their encompassed genes. Because invLOEUF is an externally derived measure of gene constraint, this approach provides an objective classification that captures relative pathogenicity while minimizing potential bias arising from estimates derived from the study sample itself

eFigure 2: *Comparison of rCNV group-associated absolute risk between SSD and SSD without comorbid childhood-onset disorders in iPSYCH2015.*

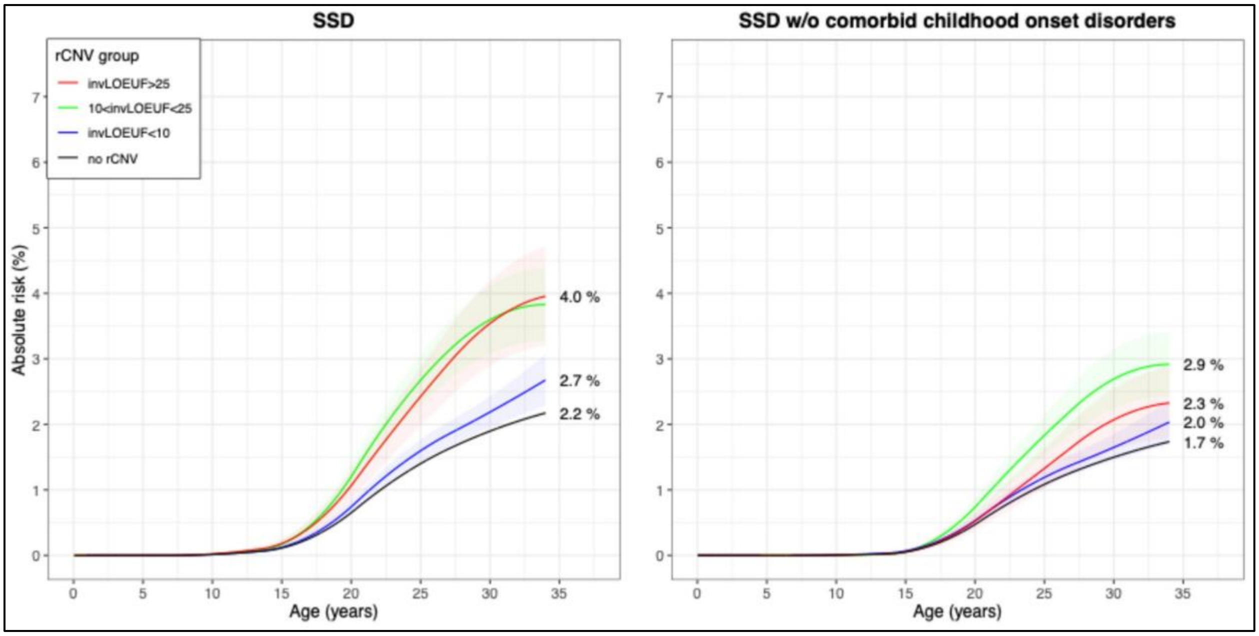

We conducted two sets of analyses to estimate absolute risks associated with each rCNV group for SSD: (i) including all individuals diagnosed with SSD and (ii) excluding individuals with comorbid childhood-onset disorders, specifically intellectual disability (ICD10: F70-F79), ASD (ICD-10: F84), and ADHD (ICD-10: F90). rCNVs were grouped by their locus' invLOEUF scores into three categories: ≤10, 10–25, and ≥25. Absolute risk curves for each group are shown in blue, green, and red, respectively, with semitransparent shading representing standard errors.

© 2026 Vaez M et al. *JAMA Psychiatry*.

eFigure 3: *Comparison of PGS group-associated absolute risk between SSD and SSD without comorbid childhood-onset disorders in iPSCYH2015.*

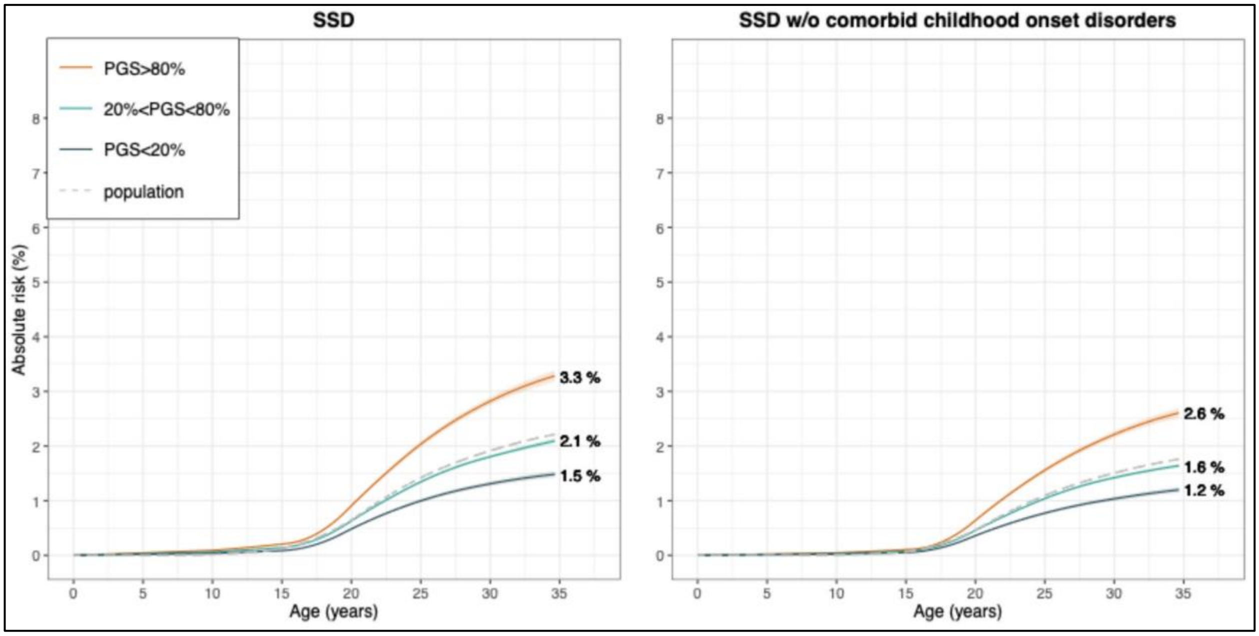

Absolute risks associated with each PGS group for SSD were estimated both including all SSD cases and those with SSD but without any comorbid childhood-onset disorders (intellectual disability [ICD-10: F70-F79], ASD [ICD-10: F84], or ADHD [ICD-10: F90]). PGS groups (i.e., PGS20%, 20%<PGS<80%, and PGS80%) are shown with absolute risk curves in light brown, green, and dark gray; shaded areas represent SE. The dashed line indicates population-average risk. Curves were smoothed with R's cobs function in from Cob's package.

© 2026 Vaez M et al. *JAMA Psychiatry*.

<u>eFigure 4:</u> *Comparison of absolute risk associated with joint rCNV and PGS groups for SSD and SSD without comorbid childhood-onset disorders in the iPSYCH2015.*

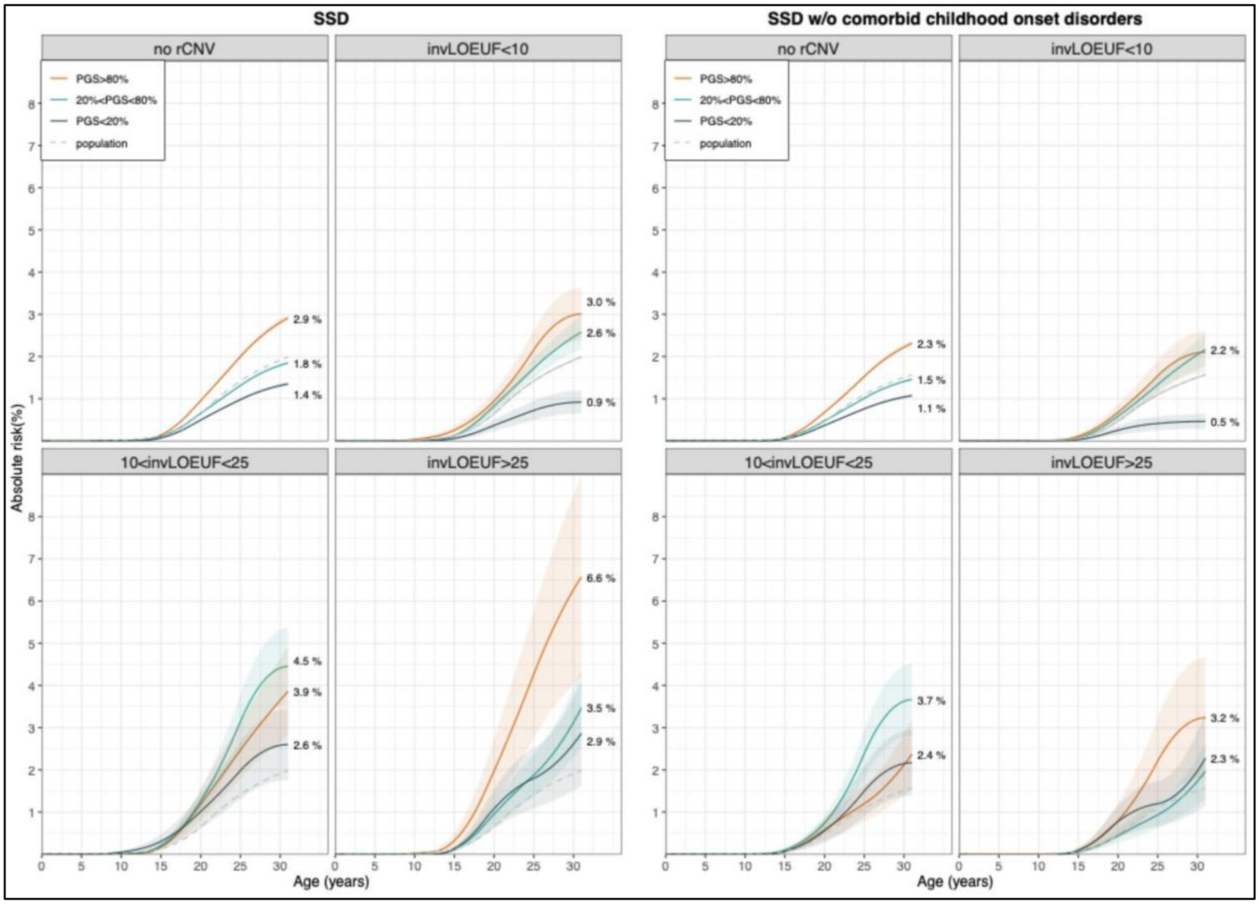

Joint absolute risks associated with rCNV and PGS group for SSD were estimated once including all the SSD cases and also for those without comorbid childhood-onset disorders (intellectual disability [ICD-10: F70-F79], ASD [ICD-10: F84], or ADHD [ICD-10: F90]). Absolute risk curves for non-carriers and rCNV carriers are shown in four panels, stratified by PGS groups (light brown, green, dark gray) with SE indicated by transparent shading. Curves are shown up to age 31 due to low carrier counts at older ages. Dashed lines indicate population-average risk. All curves were smoothed using the cobs function in R.

© 2026 Vaez M et al. *JAMA Psychiatry*.

eFigure 5: *Comparison of absolute risk estimates and proportion of at-risk individuals attributed to 6 common rCNVs and disorder-specific PGS for ASD, ADHD, SSD, and MDD in the iPSYCH2015 random Cohort.*

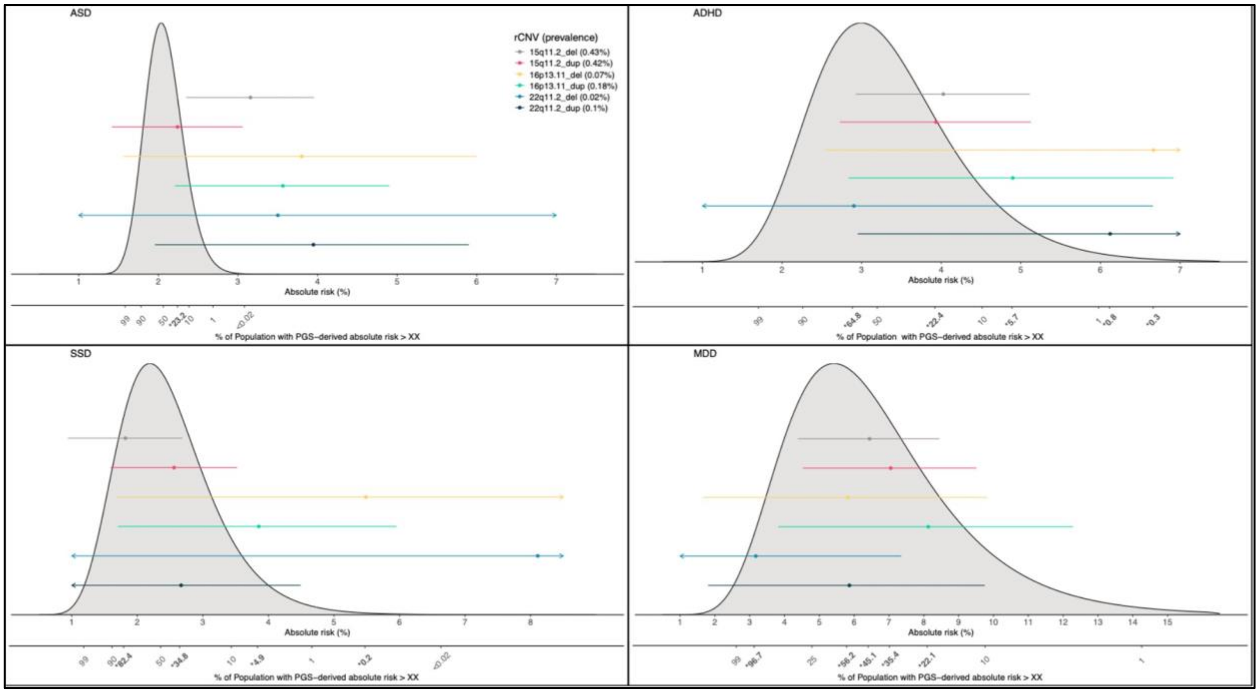

Each panel presents a comparison of derived absolute risks associated with six common rCNVs and those derived from PGS, together with the proportion of at-risk individuals identified by each genetic predictor (i.e, rCNV vs PGS). rCNV associated absolute risk with 95% CI are color-coded by rCNV. Values shown in parentheses next to each rCNV indicate their prevalence in the iPSYCH2015 random cohort. Density curves depict the distribution of PGS-derived absolute risks based on 100,000 artificially generated PGS quantiles obtained by sampling from distributions parameterized by the mean and standard deviation of disorderspecific PGS in iPSYCH2015 random cohort. Upper x-axis reports the obtained absolute risk values, and the lower x-axis indicates proportion of individuals with PGS-associated risk equal to or greater than that observed for each rCNV. Bolded labels on the lower x-axis that are marked with an asterisk denote the proportion corresponding to each comparison. (see Methods and eMethods).

© 2026 Vaez M et al. *JAMA Psychiatry*.

<u>eFigure 6:</u> Calibration analysis for disease-specific PGSs in iPSYCH2015.

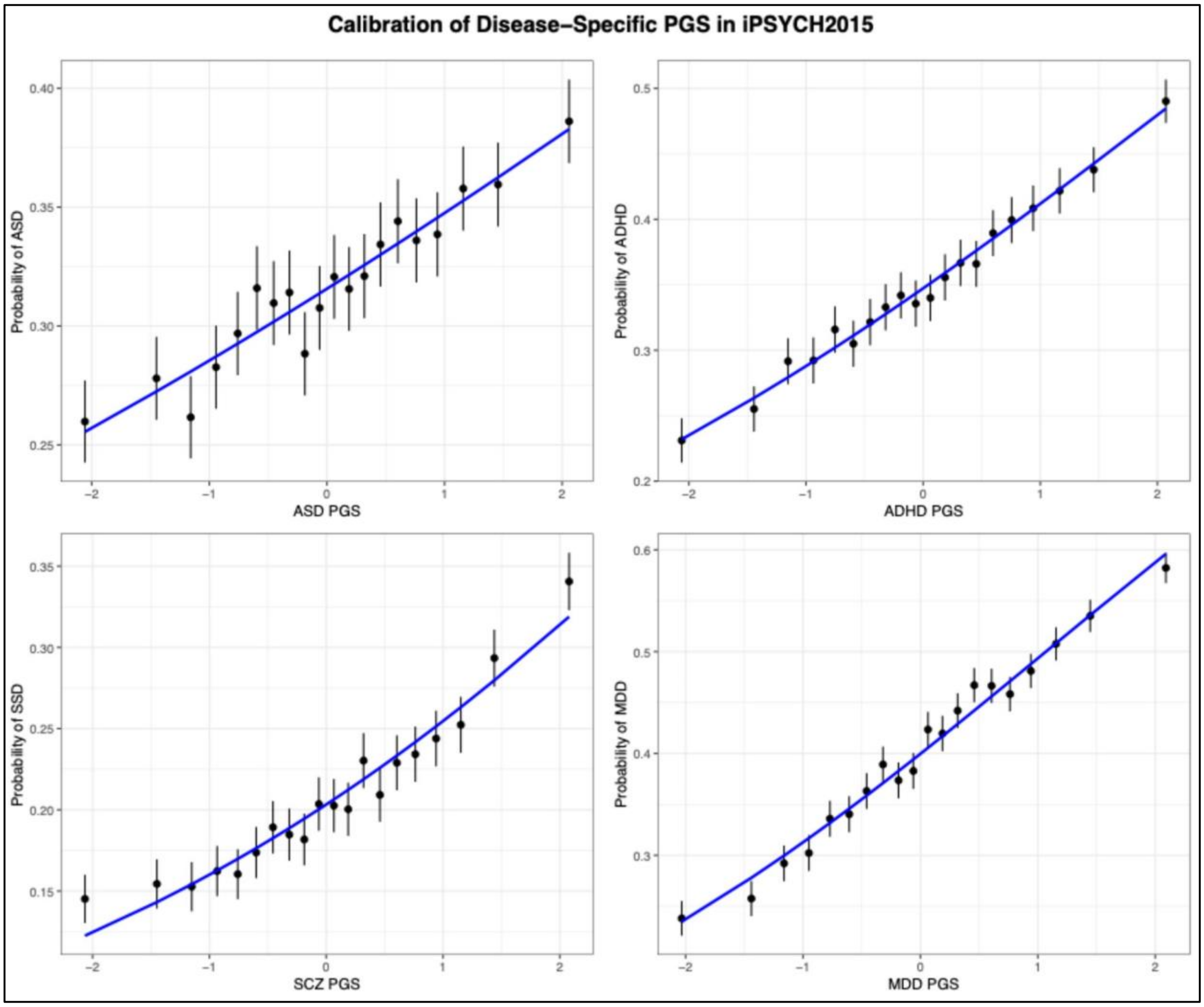

We performed disease-specific PGS calibration analysis for ASD, ADHD, SSD, and MDD presented in four panels. Observed outcome probabilities were estimated within 5% cumulative intervals of the polygenic score distribution using intercept-only logistic regression models. Expected probabilities were derived from the full logistic regression model evaluated at the mean polygenic score within each interval. X- and y-axis show probability of each diagnosis, and polygenic scores, respectively. Point estimates and terror bars represent observed probabilities and their 95% confidence intervals across 20 equal bins. Blue line shows expected probabilities for each diagnosis (supplementary Method). Observed and expected probabilities are closely aligned across the distribution, indicating adequate calibration within the precision in iPSYCH2015.

© 2026 Vaez M et al. *JAMA Psychiatry*.

<u>eFigure 7:</u> *Comparison of mean PGS between rCNV carriers and non-carriers across cases and controls.*

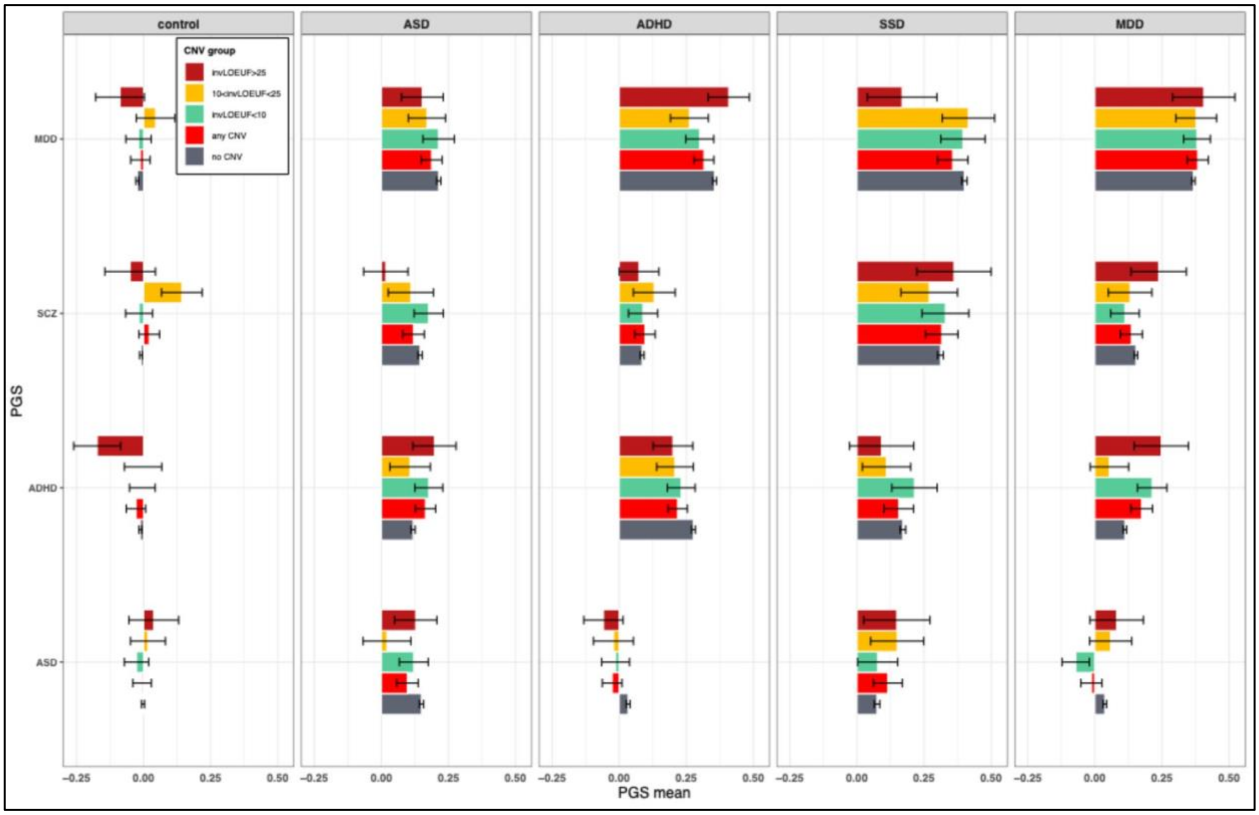

Mean PGS values for each diagnosis were compared across non-carriers, carriers of specific rCNV groups, and carriers of any rCNV, separately for ASD, ADHD, SSD, MDD, and controls in different panels. Error bars represent the standard error of the mean for each group (eTable 14). Within each panel, individuals are stratified by rCNV group status, distinguished by color. To statistically assess differences in PGS between rCNV carriers and non-carriers, we fitted a series of GLMs and linear regression models across the four diagnoses, predicting rCNV carrier status either as any rCNV or stratified by invLOEUF-defined groups (Methods & eMethods). Across all disorders, we found no evidence of significant differences in PGS between rCNV carriers and non-carriers among cases or controls (PLRT > 0.13).

© 2026 Vaez M et al. *JAMA Psychiatry*.

eFigure 8: *Comparison of disease-specific PGS among rCNV carriers and non-carriers across different diagnostic groups.*

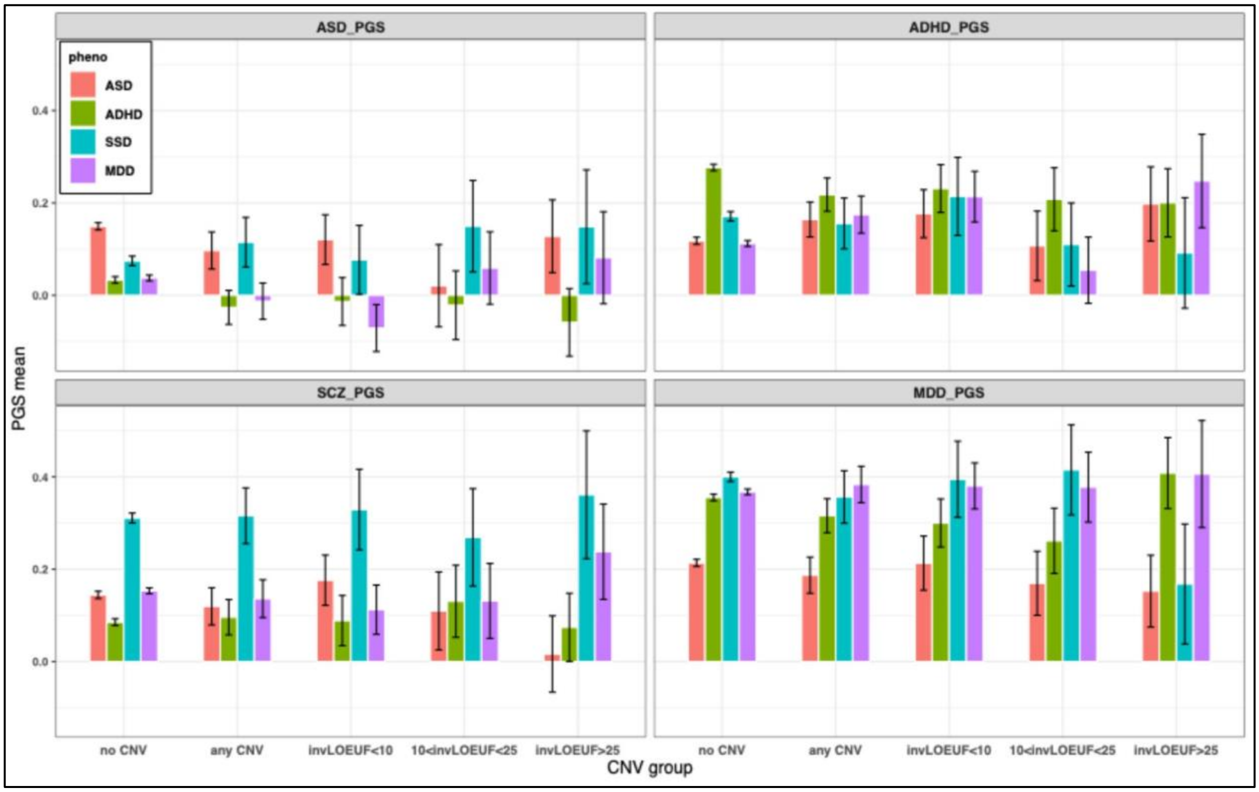

Panels show mean of disease-specific PGS for ASD, ADHD, SSD, and MDD, stratified by rCNV group and affection status (i.e., ASD, ADHD, SSD, and MDD). Error bars represent the standard error of the mean for each group. Individuals within each panel are color-coded based on their indicated diagnosis. Statistical comparisons between rCNV carriers and non-carriers were performed using GLMs and linear regression models, predicting rCNV status as any rCNV or stratified by invLOEUF-defined groups (Methods & eMethods). No significant differences in disease-specific PGS were observed between rCNV carriers and non-carriers across cases or controls (PLRT > 0.13).

© 2026 Vaez M et al. *JAMA Psychiatry*.

Supplementary tables:

eTable 1: *rCNV loci used in the study and their corresponding invLOUEF score group*

rCNV locus	Hg19 range (Chr:Mb)	invLOEUF (sum)[a]	invLOUEF group[b]	Filtered[c]
TAR	1:145.39-145.81	22.2	medium	
1q21.1	1:146.53-147.39	9.1	low	
2q11.2	2:96.74-97.68	43.8	high	
2q13	2:111.39-112.01	15.84	medium	
2q21.1	2:131.48-131.93	5.1	low	
3q29	3:195.72-197.35	41.3	high	
WBS	7:72.74-74.14	50.2	high	
7q11.23d	7:75.14-76.06	17.0	medium	yes
8p23.1	8:8.10-11.87	27.5	high	
10q11.23	10:49.39-51.06	23.5	medium	
10q23	10:82.05-88.93	39.9	high	
13q12.12	13:23.56-24.88	8.0	Low	
15q11.2	15:22.81-23.09	8.4	low	
PWAS	15:24.82-28.39	19.0	medium	
15q13.3	15:31.08-32.46	8.7	low	
15q24	15:72.90-78.15	112.3	high	yes
16p13.11	16:15.51-16.29	19.0	medium	
16p12.1	16:21.95-22.43	9.0	low	
16p11.2d	16:28.82-29.05	21.4	medium	
16p11.2	16:29.65-30.20	42.9	high	
17p12	17:14.14-15.43	6.5	low	
PLS	17:16.81-20.21	76.6	high	
17q11.2	17:29.12-30.27	26.3	high	
17q12	17:34.81-36.22	37.1	high	
22q11.2	22:18.90-20.30	50.4	high	
22q11.2b	22:20,71-21,47	24.2	medium	
22q11.2d	22:21,92-23,65	30.6	high	

© 2026 Vaez M et al. *JAMA Psychiatry*.

[a] The invLOEUF score of each locus was computed by inverting the sum of the LOEUF scores of encompassing locus genes (since lower scores indicate higher gene constraint, scores were inverted) [b] We grouped rCNV loci into three groups according to their locus-invLOEUF score (low-impact; invLOEUF<10, medium-impact; 10<invLOEUF<25, and high-impact; invLOEUF>25) [c] Loci with no carriers in the unrelated European sample were excluded.

eTable 6: Number of rCNV carriers within invLOEUF groups and collectively across cohort and cases in iPSYCH2015 case-cohort (Unrelated European sample)

invLOEUF group	iPSYCH[a]	Cohort[b]	ASD	ADHD	SSD	MDD	SSD w/o ID/ASD/ADHD [c]
Low	1486	495	319	371	148	368	107
Medium	746	229	163	187	103	176	70
High	516	131	159	173	62	94	34
Any rCNV	2748	855	641	731	313	638	211

[a] Some individuals are diagnosed with more than one condition; therefore, the number of rCNV carriers within each invLOEUF group in the entire iPSYCH does not correspond to the sum of carrier counts across cases and cohort.[b] Due to the overlap between cases and the random cohort, some carriers within the random cohort may also belong to the case samples.[c] SSD cases without any comorbid childhood-onset disorders (intellectual disability [ICD-10: F70-F79], ASD [ICD-10: F84], or ADHD [ICD-10: F90]).

eTable 7: Associated effect sizes and standard errors of rCNV-invLOEUF groups and PGS on ASD, ADHD, SSD, and MDD derived from fitted GLMs.

	rCNV groups[a]			PGS[b]		
**Diagnosis**	**β**	**SE**	**P**	**β**	**SE**	**P**
ASD	0.33	0.031	$<2.2 \times 10^{-16}$	0.14	0.010	$<2.2 \times 10^{-16}$
ADHD	0.29	0.029	$<2.2 \times 10^{-16}$	0.28	0.009	$<2.2 \times 10^{-16}$
SSD	0.25	0.039	5.66×10^{-10}	0.28	0.012	$<2.2 \times 10^{-16}$
MDD	0.04	0.034	0.27	0.38	0.009	$<2.2 \times 10^{-16}$

[a] To statistically evaluate the observed increase in absolute risk of ASD, ADHD, SSD, but not MDD, associated with rCNV groups and PGS levels, we constructed different GLM models using rCNV status based on invLOEUF groups as the linear explanatory variable for predicting each outcome separately (Supplementary Method). [b] Similarly, as we observed that an increase in PGS levels was associated with an elevated absolute risk of ADHD, ASD, SSD, and MDD, we built GLM models using PGS as the linear independent variable to predict each outcome separately. β, SE, and P correspond to the beta, standard error, and P value of the explanatory variable, respectively, derived from the summary of the fitted GLM models.

© 2026 Vaez M et al. *JAMA Psychiatry*.

eTable 9: *Model fitting results for ASD, ADHD, SSD, and MDD as a function of PGS and rCNV status (as invLOEUF groups and aggregated).*

	ASD					ADHD				
**Model**	**Genetic exposure** [a]	**df**	**Chisq** [b]	**P** [c]	**ΔR2** [d]	**Model**	**df**	**Chisq** [b]	**P** [c]	**ΔR2** [d]
0	None	3				0	3			
1	CNV_groups	4	114.80	$<2.2 \times 10^{-16}$	0.003	1	4	100.96	$<2.2 \times 10^{-16}$	0.002
2	anyCNV	4	88.58	$<2.2 \times 10^{-16}$	0.002	2	4	83.14	$<2.2 \times 10^{-16}$	0.002
3	PGS	4	199.99	$<2.2 \times 10^{-16}$	0.005	3	4	945.19	$<2.2 \times 10^{-16}$	0.022
4	PGS + CNV_groups	5	115.31	$<2.2 \times 10^{-16}$	0.007	4	5	103.45	$<2.2 \times 10^{-16}$	0.024
5	PGS + anyCNV	5	200.85	$<2.2 \times 10^{-16}$	0.007	5	5	946.24	$<2.2 \times 10^{-16}$	0.024
6	PGS×CNV_groups	8	2.78	0.42	0.007	6	8	2.81	0.42	0.024
7	PGS×anyCNV	6	0.50	0.48	0.007	7	6	1.12	0.29	0.024

	SSD					MDD [e]				
**Model**	**Genetic exposure** [a]	**df**	**Chisq** [b]	**P** [c]	**ΔR2** [d]	**Model**	**df**	**Chisq** [a]	**P** [c]	**ΔR2** [d]
0	None	3				0	3			
1	CNV_groups	4	38.47	2.25×10^{-8}	0.001	1	4	2.76	0.43	5×10^{-5}
2	anyCNV	4	27.40	1.65×10^{-7}	0.009	2	4	1.48	0.22	3×10^{-5}
3	PGS	4	560.36	$<2.2 \times 10^{-16}$	0.0180	3	4	1798.07	$<2.2 \times 10^{-16}$	0.03
4	PGS + CNV_groups	5	36.54	5.75×10^{-8}	0.019	4				
5	PGS + anyCNV	5	558.40	$<2.2 \times 10^{-16}$	0.019	5				
6	PGS×CNV_groups	8	3.34	0.34	0.019	6				
7	PGS×anyCNV	6	0.90	0.34	0.019	7				

We fitted different GLMs to assess the combined and interactive effects of rCNV and PGS on ASD, ADHD, SSD, and MDD using rCNVs divided into three invLOEUF groups and rCNV in an aggregated form (i.e., any rCNV vs. no rCNV) (see Method). LRTs were used to compare each model with its nested model. The null model included sex, age at the end of follow-up, and the genotyping array as the independent variables. [a] rCNV and PGS group status were added stepwise to the null model. [b] Chisq and [c] P are derived from the likelihood ratio tests. [d] Niekerk's pseudo-R2 was calculated for each model, and ΔR^2 was derived by subtracting the pseudo-R2 of each full model from the pseudo-R2 of the nested model. [e] Since no significant effect of rCNV was found on the risk of MDD, we did not report the results of the additive and interactive effect of rCNV with PGS in this case.

© 2026 Vaez M et al. *JAMA Psychiatry*.

eTable 10: *Model fitting results for schizophrenia (SCZ) and SSD without comorbid childhood-onset disorders as a function of PGS and rCNV status (using rCNVs as invLOEUF groups and aggregated)*

SCZ						SCZ w/o ID/ASD/ADHD				
**Model**	**Genetic exposure** [a]	**df**	**Chisq** [b]	**P** [c]	**ΔR2** [d]	**Model**	**df**	**Chisq** [b]	**P** [c]	**ΔR2** [d]
0	None	3				0	3			
1	CNV_groups	4	27.75	4.09×10^{-6}	0.001	1	4	14.45	0.0023	0.0005
2	anyCNV	4	21.21	4.12×10^{-6}	0.001	2	4	10.19	0.0014	0.0004
3	PGS	4	386.13	$<2.2 \times 10^{-16}$	0.0180	3	4	440.40	$<2.2 \times 10^{-16}$	0.0160
4	PGS + CNV_groups	5	25.75	1.07×10^{-5}	0.0190	4	5	14.17	0.0027	0.0162
5	PGS + anyCNV	5	384.41	$<2.2 \times 10^{-16}$	0.0190	5	5	439.81	$<2.2 \times 10^{-16}$	0.0160
6	PGS×CNV_groups	8	3.80	0.28	0.0190	6	8	3.89	0.27	0.0163
7	PGS×anyCNV	6	2.12	0.14	0.0190	7	6	2.11	0.14	0.0160

In addition to the main analysis of assessing the joint effect of rCNV and PGS on schizophrenia spectrum disorder (SSD; ICD-10: F20-29) by GLMs, we performed similar analyses using both the narrow definition for schizophrenia (SCZ; ICD-10: F20) as well as SSD cases without comorbid childhood-onset disorders, including intellectual disability (ICD-10: F70-F79), ASD (ICD-10: F84), or ADHD (ICD-10: F90). Likelihood ratio tests were used to compare each model with its nested model. The null model included sex, age at the end of follow-up, and the genotyping array as independent variables. rCNV and PGS were added in a stepwise manner to the null model. [a] *"anyCNV" and "rCNV_group" represent rCNVs in aggregated form and rCNV invLOEUF groups, respectively.* [b] *Chisq, and* [c] *P are derived from the LRTs.* [d] *Nagelkerke's R2 was calculated for each model, and ΔR^2 was derived by subtracting the R2 of each full model from the R2 of the subsequent nested model.*

eTable 13: *CNV×PRS Interaction on ASD, ADHD, and SSD for rCNV-invLOEUF groups, aggregated rCNVs, and 9 common individual rCNVs.*

ASD

CNV× ASD-PGS	β	SE	P	CNV× ADHD-PGS	β	SE	P
Low invLOEUF	0.02	0.08	0.84	Low invLOEUF	-0.08	0.07	0.29
Medium invLOEUF	-0.18	0.11	0.09	Medium invLOEUF	-0.10	0.11	0.34
High invLOEUF	-0.01	0.13	0.94	High invLOEUF	0.12	0.13	0.37
Any CNV	-0.04	0.06	0.48	Any CNV	-0.06	0.05	0.29
TAR _dup	-0.31	0.29	0.29	TAR _dup	-0.03	0.27	0.91
1q21.1_dup	-0.16	0.30	0.60	1q21.1_dup	-0.11	0.25	0.66
15q11.2_del	-0.15	0.28	0.60	15q11.2_del	0.03	0.27	0.91
15q11.2_dup	0.05	0.30	0.86	15q11.2_dup	0.33	0.35	0.34
16p13.11_del	-0.10	0.28	0.73	16p13.11_del	0.28	0.34	0.42
16p13.11_dup	-0.03	0.18	0.87	16p13.11_dup	-0.51	0.18	**0.0057***
16p12.1_del	0.18	0.16	0.28	16p12.1_del	-0.07	0.12	0.58
16p11.2_dup	0.01	0.14	0.95	16p11.2_dup	-0.09	0.13	0.46
22q11.2_dup	-0.01	0.27	0.96	22q11.2_dup	-0.07	0.23	0.76

© 2026 Vaez M et al. *JAMA Psychiatry*.

SSD				*SSD w/o ID/ASD/ADHD* [a]			
**CNV× SCZ-PGS**	β	**SE**	**P**	**CNV× SCZ-PGS**	β	**SE**	**P**
Low invLOEUF	-0.05	0.09	0.57	Low invLOEUF	-0.04	0.11	0.70
Medium invLOEUF	-0.18	0.12	0.12	Medium invLOEUF	-0.26	0.14	0.052
High invLOEUF	0.14	0.17	0.41	High invLOEUF	-0.004	0.21	0.98
Any CNV	-0.06	0.07	0.34	Any CNV	-0.12	0.08	0.14
TAR _dup	0.23	0.30	0.46	TAR _dup	-0.70	0.45	0.12
1q21.1_dup	-0.53	0.28	0.06	1q21.1_dup	0.005	0.39	0.99
15q11.2_del	-0.44	0.37	0.24	15q11.2_del	-0.20	0.23	0.39
15q11.2_dup	-0.02	0.35	0.96	15q11.2_dup	-0.01	0.19	0.94
16p13.11_del	-0.47	0.34	0.17	16p13.11_del	-1.93	0.79	**0.0148***
16p13.11_dup	0.04	0.25	0.88	16p13.11_dup	0.02	0.30	0.94
16p12.1_del	0.03	0.17	0.84	16p12.1_del	-0.41	0.32	0.20
16p11.2_dup	-0.07	0.18	0.71	16p11.2_dup	-0.18	0.45	0.68
22q11.2_dup	-0.09	0.32	0.78	22q11.2_dup	0.05	0.33	0.87

*Interaction terms are attributed to the fitted GLMs for assessing the interactive effects of rCNV and PGS on ASD, ADHD, and SSD (see method; eTable 8 a-c). We tested rCNV×PGS using rCNV groups (divided based on their invinvLOEUF scores), common individual rCNVs, and aggregated rCNVs (any rCNV vs. no rCNV). Interaction effects on MDD are not reported since we found no indication of a significant effect of rCNVs on MDD. [a] We conducted a similar analysis for SSD, restricting the cases to individuals with SSD who did not have comorbid childhood-onset disorders, including intellectual disability (ICD-10: F70-F79), ASD (ICD-10: F84), or ADHD (ICD-10: F90). * Coefficients for interaction terms with statistically significant post hoc p-values ($p < 0.05$) are shown in bold.*

eTable 14: *Number of carriers of the common rCNVs in iPSYCH2015 across cohort and case samples (Unrelated European sample).*

**rCNV**	**iPSYCH** [a]	**Cohort** [b]	**ASD**	**ADHD**	**SSD**	**MDD**	***SSD w/o ID/ASD/ADHD*** [c]
TAR_dup	131	42	26	40	10	37	6
1q21.1_dup	103	26	41	32	13	17	8
15q11.2_del	484	155	114	123	34	123	22
15q11.2_dup	448	157	72	110	48	116	39
16p13.11_del	105	27	22	32	19	20	8
16p1311_dup	226	66	55	59	27	58	20
16p12.1_del	128	45	21	36	13	30	8
16p11.2_dup	146	34	39	61	12	33	10
22q11.2_dup	127	37	35	42	14	24	10

In addition to the main analyses of assessing the joint effects of PGS and rCNVs (i.e., in the form of invLOEUF groups and aggregated) on ASD, ADHD, SSD, and MDD using GLMs, we conducted similar analyses using common individual rCNVs that had over $1/1000$ carriers in the entire sample. [a] Some individuals are diagnosed with more than one condition; therefore, the number of rCNV carriers in the entire iPSYCH does not correspond to the sum of the carrier counts across columns of cases and cohort. [b] Due to the overlap between cases and the random cohort, some carriers within the random cohort may be also part of the case samples. [c] We conducted a similar analysis for SSD, restricting the cases to individuals with SSD who did not have comorbid childhood-onset disorders, including intellectual disability (ICD-10: F70-F79), ASD (ICD-10: F84), or ADHD (ICD-10: F90).

© 2026 Vaez M et al. *JAMA Psychiatry*.

eTable 15: *Comparison of odds ratios (OR) and risk ratios (RR) attributed to diseasespecific PGS across ASD, ADHD, SSD, and MDD.*

Diagnosis	OR (95% CI) [a]	RR (95% CI) [b]
ASD	1.1230 (1.0978- 1.1489)	1.1202 (1.1201-1.1203
ADHD	1.3182 (1.2926-1.3444)	1.3053 (1.3049-1.3057)
SSD	1.3462 (1.3160-1.3172)	1.3355 (1.3351-1.3358)
MDD	1.4684 (1.4438-1.4939)	1.4297 (1.4287-1.4307)

[a] Odds ratio (OR) and 95% confidence interval corresponding to a 1 SD increase in the standardized PGS for each diagnosis, derived from fitted generalized linear models (GLMs). [b] Risk ratio (RR) and 95% confidence interval attributable to each disorder-specific PGS, calculated using the estimated PGS effect (β_{pgs}) from the fitted GLMs and the population-average absolute risk for each disorder (see eMethods).

eTable 16: *Proportion of individuals whose PGS-derived absolute risk exceeds the risk associated with rCNV groups across ASD, ADHD, SSD, and MDD.*

	ASD			ADHD		
rCNV group	Absolute Risk [a]		% of At-risk individuals in PGS distribution [b]	Absolute Risk[a]		% of At-risk individuals in PGS distribution [b]
Low invLOEUF	LCI	2.44	7.10 (6.31-11.06)	LCI	3.33	44.60 (44.60-44.97)
	Estimate	2.98	0.06 (0-0.34)	Estimate	4.03	19.9 (18.13-21.60)
	UCI	3.52	0 (0-0.02)	UCI	4.71	7.57 (6.08-9.08)
Middle invLOEUF	LCI	2.44	6.71(3.06-10.60)	LCI	3.36	43.34 (42.82-43.79)
	Estimate	3.40	0.0	Estimate	4.36	12.61 (10.82-14.30)
	UCI	4.36	0 (0-0)	UCI	5.36	2.79 (1.95-3.74)
High invLOEUF	LCI	4.03	0 (0-0)	LCI	4.73	7.45 (5.97-8.94)
	Estimate	5.36	0 (0-0)	Estimate	6.34	0.55 (0.31-0.90)
	UCI	6.68	0 (0-0)	UCI	7.92	0.04 (0-0.08)
	SSD			MDD		
Low invLOEUF	LCI	2.01	72.28 (73.94-70.82)	LCI	5.33	65.53 (66.26-64.86)
	Estimate	2.81	28.30 (26.68-29.73)	Estimate	6.60	42.37 (42.00-42.71)
	UCI	3.61	7.54 (5.96-9.14)	UCI	7.85	25.03 (23.98-26.01)
Middle invLOEUF	LCI	2.87	26.14 (24.42-27.68)	LCI	4.89	74.00 (75.02-73.05)
	Estimate	4.02	3.57 (2.53-4.73)	Estimate	6.93	37.28 (36.67-37.83)
	UCI	5.16	0.39 (0.19-0.68)	UCI	8.92	15.24 (14.07-16.36)

© 2026 Vaez M et al. *JAMA Psychiatry*.

High invLOEUF	LCI	2.58	38.90 (37.99-39.69)	LCI	3.84	90.58 (91.63-89.54)
	Estimate	4.15	2.78 (1.89-3.79)	Estimate	9.88	9.55 (8.50-10.60)
	UCI	5.70	0.13 (0.05-0.27)	UCI	18.52	0.12 (0.07-0.18)

Proportion of the population wit PGS–derived absolute risk equal to or exceeding the absolute risk associated with rCNVs. Population absolute risk and rCNV-associated risk were estimated using weighted survival models, and PGS effect sizes were obtained from covariate-adjusted logistic regression. An artificial population PGS distribution was constructed by sampling 100,000 representative quantiles based on the mean and standard deviation observed in the random subcohort. Expected absolute risk was calculated for each quantile, and the minimum PGS quantile at which PGS-derived risk exceeded rCNV-associated risk was identified. Reported values represent the upper-tail cumulative probability of the PGS distribution, with uncertainty assessed using confidence interval bounds of the PGS effect estimates. (see eMethods). [a] Absolute risk and its 95% confidence interval associated with each rCNV group (three rows). [b] Proportion of individuals with equal or higher PGS-derived absolute risk than that of the corresponding rCNV group; 95% confidence intervals for the estimated proportion are shown in parentheses.

eTable 17: *Proportion of individuals whose PGS-derived absolute risk exceeds the risk associated with 6 common rCNVs across ASD, ADHD, SSD, and MDD.*

	ASD			ADHD		
**rCNV**	**Absolute Risk [a]**		**% of At-risk individuals in PGS distribution [b]**	**Absolute Risk[a]**		**% of At-risk individuals in PGS distribution [b]**
**15q11.2 deletion**	LCI	2.35	12.43 (7.46-16.82)	LCI	2.93	63.55 (64.58-62.66)
	Estimate	6.24	0 (0-0)	Estimate	4.03	19.92 (18.11-21.58)
	UCI	14.22	0 (0-0)	UCI	5.11	4.12 (3.04-5.28)
**15q11.2 duplication**	LCI	1.42	99.99 (99.80-99.71)	LCI	2.73	73.00 (74.58-71.59)
	Estimate	2.24	23.21 (18.01-27.10)	Estimate	3.93	22.41 (20.66-24.00)
	UCI	3.06	0.02 (0 -0.19)	UCI	5.13	4.02 (2.96-5.17)
**16p13.11 deletion**	LCI	1.56	99.31 (99.89-97.98)	LCI	2.54	81.00 (82.83-79.32)
	Estimate	3.80	0 (0-0)	Estimate	6.67	0.32 (0.16-0.55)
	UCI	6.00	0 (0-0)	UCI	10.62	0
**16p13.11 duplication**	LCI	2.21	27.13 (22.33-30.59)	LCI	2.84	67.96 (69.28-66.81)
	Estimate	3.56	0 (0-0.02)	Estimate	4.90	5.73 (4.43-7.08)
	UCI	4.90	0 (0-0)	UCI	6.92	0.21 (0.10-0.38)
**22q11.2 deletion**	LCI	0	100 (100-100)	LCI	0	100 (100-100)
	Estimate	3.50	0	Estimate	2.90	64.76 (65.87-63.79)
	UCI	7.70	0	UCI	6.66	0.30 (0.16-0.55)

© 2026 Vaez M et al. *JAMA Psychiatry*.

**22q11.2 duplication**	LCI	1.96	67.88 (71.92-65.06)	LCI	2.95	62.42 (63.37-61.60)
	Estimate	3.95	0	Estimate	6.12	0.80 (0.46-1.24)
	UCI	5.90	0	UCI	9.19	0
		**SSD**			**MDD**	
**15q11.2 deletion**	LCI	0.94	99.93 (99.97-99.84)	LCI	4.39	82.65 (83.82-81.53)
	Estimate	1.82	82.38 (84.35-80.58)	Estimate	6.44	45.09 (44.85-45.31)
	UCI	2.69	33.91 (32.63-35.02)	UCI	8.45	19.08 (17.93-2017)
**15q11.2 duplication**	LCI	1.59	91.81 (93.45-90.17)	LCI	4.53	80.39 (81.55-79.31)
	Estimate	2.56	40.13 (39.31-40.83)	Estimate	7.05	35.42 (34.73-36.05)
	UCI	3.52	8.88 (7.18-10.56)	UCI	9.51	11.46 (10.35-12.54)
**16p13.11 deletion**	LCI	1.69	88.05 (89.93-86.26)	LCI	1.66	99.99 (99.99-99.98)
	Estimate	5.49	0.20 (0.09-0.38)	Estimate	5.82	56.21 (56.51-55.93)
	UCI	9.14	0	UCI	9.81	9.87 (8.80-10.92)
**16p13.11 duplication**	LCI	1.70	87.58 (89.48-85.78)	LCI	3.82	90.80 (91.84-89.77)
	Estimate	3.85	4.87 (3.61-6.21)	Estimate	8.13	22.11 (21.00-23.15)
	UCI	5.96	0.08 (0.04-0.17)	UCI	12.28	2.80 (2.25-3.41)
**22q11.2 deletion**	LCI	0	100 (100-100)	LCI	0	100 (100-100)
	Estimate	8.11	0 (0-0)	Estimate	3.18	96.71 (97.33-96.05)
	UCI	19.37	0 (0-0)	UCI	7.35	31.22 (3.37-32.01)
**22q11.2 duplication**	LCI	0.81	99.99 (100-99.97)	LCI	1.81	99.97 (99.98-99.94)
	Estimate	2.67	34.77 (33.55-35.82)	Estimate	5.86	55.47 (55.74-55.23)
	UCI	4.49	1.44 (0.89-2.13)	UCI	9.75	10.16 (9.09-11.22)

We additionally conducted the same analyses as described for the previous table, restricting the comparison to six individual rCNVs. Further methodological details are provided in the caption of eTable 13a.

© 2026 Vaez M et al. *JAMA Psychiatry*.

eTable 19: *LRT results for comparison of disorder-specific PGSs between rCNV carriers and non-carriers across ASD, ADHD, SSD, and MDD.*

	ASD			ADHD		
**rCNV**	**df** [a]	**Chisq** [b]	**p** [c]	**df** [a]	**Chisq** [b]	**p** [c]
**invLOEUF groups**	1	0	0.97	1	0.15	0.70
**Any rCNV**	1	0.08	0.78	1	0	0.9
	**SSD**			**MDD**		
**invLOEUF groups**	1	2.25	0.13	1	0.44	0.51
**Any rCNV**	1	2.12	0.14	1	0.68	0.41

We tested whether the average disorder-specific PGS differs between rCNV carriers and non-carriers within the case-control of each diagnostic group. To assess this for overall rCNV carriers (i.e., carriers of any rCNV), we fitted a series of GLMs with rCNV carrier status as the outcome. The nested model included sex, age, genotyping array, and 20 PCs as covariates. The full model included the same covariates plus the PGS of the tested disorder as an additional predictor. Model fit was compared using LRTs. We repeated this analysis by redefining carrier status based on rCNV-invLOEUF group membership, employing multinomial regression models to account for the categorical structure (see Methods and Supplementary Methods for details). The results from LRTs comparing full and nested models are presented separately for each disorder. [a] degree of freedom; [b] chi-square test; and [c] pvalue derived from LRTs.

eTable 20: *Results from LRTs comparing overall psychiatric, behavioural, and somatic PGS profiles between rCNV carriers and non-carriers.*

		ASD			ADHD		
**rCNV**	**PGS profile** [a]	**df** [b]	**Chisq** [c]	**p** [d]	**df** [b]	**Chisq** [c]	**p** [d]
**Any rCNV**	psychiatric	4	2.02	0.73	4	3.91	0.41
	Psychiatric + behavioural	5	17.29	0.0040	5	13.65	0.0180
	Psychiatric + behavioural+somatic	4	2.32	0.68	4	1.46	0.83
**invLOEUF groups**	Psychiatric + behavioural + somatic	39	47.24	0.17	39	49.57	0.12
		**SSD**			**MDD**		
**Any rCNV**	psychiatric	4	0.30	0.99	4	1.02	0.91
	psychiatric + behavioural	5	10.76	0.06	5	7.52	0.18
	psychiatric +behavioural + somatic	4	9.57	0.0480	4	4.89	0.30
**invLOEUF groups**	psychiatric + behavioural + somatic	39	41.31	0.37	39	36.30	0.59

We evaluated whether rCNV carriers exhibit a different overall polygenic profile, constructed from 13 distinct phenotypes, compared to non-carriers. To assess this among overall rCNV carriers (i.e, any rCNV), several GLMs were used to predict the rCNV carrier status as the outcome. Each analysis began with a base model comprising sex, age, genotyping array, and 20 PCs. PGSs for five psychiatric, five behavioral, and three somatic phenotypes were then incorporated sequentially into the model as additional covariates, with model improvements evaluated using LRTs. Additionally, a similar analysis was performed to assess polygenic differences among rCNV carriers stratified by invLOEUF-defined groups. For this, we employed two multinomial logistic regression

© 2026 Vaez M et al. *JAMA Psychiatry*.

models: a base model that included the same primary covariates, and a full model that additionally incorporated all 13 PGSs. [a] psychiatric: ASD, ADHD, SCZ, MDD, and BPD; behavioural: risk-taking behavior, neuroticism, alcoholism, intelligence, and educational attainment; somatic traits: diabetes, coronary artery disease, and BMI. [b] degrees of freedom; [c] chi-square test; and [d] pvalue corresponding to LRTs. Among all LRT results, three comparisons yielded significant p-values (P < 0.05). However, after applying false discovery rate (FDR) correction to the p-values derived from the summary of each fitted model, none remained significant. The summary of results from fitted GLMs, including FDR-adjusted p-values, is presented in the next table (see eTable 17).

© 2026 Vaez M et al. *JAMA Psychiatry*.

eReferences:

1. McCarthy S, Das S, Kretzschmar W, et al. A reference panel of 64,976 haplotypes for genotype imputation. *Nat Genet*. 2016;48(10):1279-1283. doi:10.1038/ng.3643

2. Bybjerg-Grauholm J, Bøcker Pedersen C, Bækvad-Hansen M, et al. The iPSYCH2015 Case-Cohort sample: updated directions for unravelling genetic and environmental architectures of severe mental disorders. Epidemiology. Preprint posted online December 2, 2020. doi:10.1101/2020.11.30.20237768

3. The 1000 Genomes Project Consortium, Corresponding authors, Auton A, et al. A global reference for human genetic variation. *Nature*. 2015;526(7571):68-74. doi:10.1038/nature15393

4. Pedersen CB, Bybjerg-Grauholm J, Pedersen MG, et al. The iPSYCH2012 case–cohort sample: new directions for unravelling genetic and environmental architectures of severe mental disorders. *Mol Psychiatry*. 2018;23(1):6-14. doi:10.1038/mp.2017.196

5. Pedersen CB. The Danish Civil Registration System. *Scand J Public Health*. 2011;39(7_suppl):22-25. doi:10.1177/1403494810387965

6. Manichaikul A, Mychaleckyj JC, Rich SS, Daly K, Sale M, Chen WM. Robust relationship inference in genome-wide association studies. *Bioinformatics*. 2010;26(22):2867-2873. doi:10.1093/bioinformatics/btq559

7. Appadurai V, Bybjerg-Grauholm J, Krebs MD, et al. Accuracy of haplotype estimation and whole genome imputation affects complex trait analyses in complex biobanks. *Commun Biol*. 2023;6(1):101. doi:10.1038/s42003-023-04477-y

8. Vaez M, Montalbano S, Calle Sánchez X, et al. Population-Based Risk of Psychiatric Disorders Associated With Recurrent Copy Number Variants. *JAMA Psychiatry*. Published online June 26, 2024. doi:10.1001/jamapsychiatry.2024.1453

9. Malhotra D, Sebat J. CNVs: Harbingers of a Rare Variant Revolution in Psychiatric Genetics. *Cell*. 2012;148(6):1223-1241. doi:10.1016/j.cell.2012.02.039

10. Rees E, Kendall K, Pardiñas AF, et al. Analysis of Intellectual Disability Copy Number Variants for Association With Schizophrenia. *JAMA Psychiatry*. 2016;73(9):963. doi:10.1001/jamapsychiatry.2016.1831

11. Gudmundsson OO, Walters GB, Ingason A, et al. Attention-deficit hyperactivity disorder shares copy number variant risk with schizophrenia and autism spectrum disorder. *Transl Psychiatry*. 2019;9(1):258. doi:10.1038/s41398-019-0599-y

12. Kendall KM, Rees E, Bracher-Smith M, et al. Association of Rare Copy Number Variants With Risk of Depression. *JAMA Psychiatry*. 2019;76(8):818. doi:10.1001/jamapsychiatry.2019.0566

© 2026 Vaez M et al. *JAMA Psychiatry*.

13. Rees E, Walters JTR, Georgieva L, et al. Analysis of copy number variations at 15 schizophrenia-associated loci. *Br J Psychiatry*. 2014;204(2):108-114. doi:10.1192/bjp.bp.113.131052

14. Marshall CR, Howrigan DP, Merico D, et al. Contribution of copy number variants to schizophrenia from a genome-wide study of 41,321 subjects. *Nat Genet*. 2017;49(1):27-35. doi:10.1038/ng.3725

15. Rehm HL, Berg JS, Brooks LD, et al. ClinGen — The Clinical Genome Resource. *N Engl J Med*. 2015;372(23):2235-2242. doi:10.1056/NEJMsr1406261

16. Karczewski KJ, Francioli LC, Tiao G, et al. The mutational constraint spectrum quantified from variation in 141,456 humans. *Nature*. 2020;581(7809):434-443. doi:10.1038/s41586020-2308-7

17. Autism Spectrum Disorder Working Group of the Psychiatric Genomics Consortium, BUPGEN, Major Depressive Disorder Working Group of the Psychiatric Genomics Consortium, et al. Identification of common genetic risk variants for autism spectrum disorder. *Nat Genet*. 2019;51(3):431-444. doi:10.1038/s41588-019-0344-8

18. Demontis D, Walters GB, Athanasiadis G, et al. Genome-wide analyses of ADHD identify 27 risk loci, refine the genetic architecture and implicate several cognitive domains. *Nat Genet*. 2023;55(2):198-208. doi:10.1038/s41588-022-01285-8

19. Adams MJ, Streit F, Meng X, et al. Trans-ancestry genome-wide study of depression identifies 697 associations implicating cell types and pharmacotherapies. Cell. 2025;188(3):640-652.e9. doi:10.1016/j.cell.2024.12.002

20. Trubetskoy V, Pardiñas AF, Qi T, et al. Mapping genomic loci implicates genes and synaptic biology in schizophrenia. *Nature*. 2022;604(7906):502-508. doi:10.1038/s41586-022-04434-5

21. Mullins N, Forstner AJ, O'Connell KS, et al. Genome-wide association study of more than 40,000 bipolar disorder cases provides new insights into the underlying biology. *Nat Genet*. 2021;53(6):817-829. doi:10.1038/s41588-021-00857-4

22. Sanchez-Roige S, Palmer AA, Fontanillas P, et al. Genome-Wide Association Study MetaAnalysis of the Alcohol Use Disorders Identification Test (AUDIT) in Two Population-Based Cohorts. *AJP*. 2019;176(2):107-118. doi:10.1176/appi.ajp.2018.18040369

23. 23and Me Research Team, eQTLgen Consortium, International Cannabis Consortium, et al. Genome-wide association analyses of risk tolerance and risky behaviors in over 1 million individuals identify hundreds of loci and shared genetic influences. *Nat Genet*. 2019;51(2):245-257. doi:10.1038/s41588-018-0309-3

24. 23andMe Research Team, Nagel M, Jansen PR, et al. Meta-analysis of genome-wide association studies for neuroticism in 449,484 individuals identifies novel genetic loci and pathways. *Nat Genet*. 2018;50(7):920-927. doi:10.1038/s41588-018-0151-7

25. Savage JE, Jansen PR, Stringer S, et al. Genome-wide association meta-analysis in

© 2026 Vaez M et al. *JAMA Psychiatry*.

269,867 individuals identifies new genetic and functional links to intelligence. *Nat Genet*. 2018;50(7):912-919. doi:10.1038/s41588-018-0152-6

26. Okbay A, Wu Y, Wang N, et al. Polygenic prediction of educational attainment within and between families from genome-wide association analyses in 3 million individuals. *Nat Genet*. 2022;54(4):437-449. doi:10.1038/s41588-022-01016-z

27. Yengo L, Sidorenko J, Kemper KE, et al. Meta-analysis of genome-wide association studies for height and body mass index in ∼700000 individuals of European ancestry. *Hum Mol Genet*. 2018;27(20):3641-3649. doi:10.1093/hmg/ddy271

28. Suzuki K, Hatzikotoulas K, Southam L, et al. Multi-ancestry genome-wide study in >2.5 million individuals reveals heterogeneity in mechanistic pathways of type 2 diabetes and complications. *medRxiv*. Published online March 31, 2023:2023.03.31.23287839. doi:10.1101/2023.03.31.23287839

29. Aragam KG, Jiang T, Goel A, et al. Discovery and systematic characterization of risk variants and genes for coronary artery disease in over a million participants. *Nat Genet*. 2022;54(12):1803-1815. doi:10.1038/s41588-022-01233-6

30. Sullivan PF, Agrawal A, Bulik CM, et al. Psychiatric Genomics: An Update and an Agenda. *Am J Psychiatry*. 2018;175(1):15-27. doi:10.1176/appi.ajp.2017.17030283

31. The International HapMap 3 Consortium. Integrating common and rare genetic variation in diverse human populations. *Nature*. 2010;467(7311):52-58. doi:10.1038/nature09298

32. Lloyd-Jones LR, Zeng J, Sidorenko J, et al. Improved polygenic prediction by Bayesian multiple regression on summary statistics. *Nat Commun*. 2019;10(1):5086. doi:10.1038/s41467-019-12653-0

33. Therneau T. A Package for Survival Analysis in R. R package version 3.8-3, https://CRAN.R-project.org/package=survival. Published online 2024. https://cran.rproject.org/package=survival

34. Barlow WE, Ichikawa L, Rosner D, Izumi S. Analysis of Case-Cohort Designs. *Journal of Clinical Epidemiology*. 1999;52(12):1165-1172. doi:10.1016/S0895-4356(99)00102-X

35. Ng PT, Maechler M. cobs: Constrained B-Splines (Sparse Matrix Based). Published online April 28, 2002:1.3-9-1. doi:10.32614/CRAN.package.cobs

36. Signorell A. DescTools: Tools for Descriptive Statistics. R package version 0.99.56. Published online 2024. https://github.com/andrisignorell/desctools

37. Dybdahl Krebs M, Appadurai V, Georgii Hellberg KL, et al. The relationship between genotype- and phenotype-based estimates of genetic liability to psychiatric disorders, in practice and in theory. The American Journal of Human Genetics. 2026;113(1):184-201. doi:10.1016/j.ajhg.2025.11.016

38. Lee SH, Goddard ME, Wray NR, Visscher PM. A Better Coefficient of Determination for Genetic Profile Analysis. Genetic Epidemiology. 2012;36(3):214-224. doi:10.1002/gepi.21614

© 2026 Vaez M et al. *JAMA Psychiatry*.

39. Venables WN, Ripley BD. *Modern Applied Statistics with S*. Fourth. Springer; 2002. https://www.stats.ox.ac.uk/pub/MASS4/

© 2026 Vaez M et al. *JAMA Psychiatry*.
