# Supplementary material for: Recurrent Copy Number Variants and Psychiatric Outcomes in the Context of Polygenic Scores
Source: JAMA Psychiatry. 2026 May 27;83(8):827–36. doi: 10.1001/jamapsychiatry.2026.1064 (PMC13217261; doi:10.1001/jamapsychiatry.2026.1064)
Supplement: Supplement 3. — Nonauthor Collaborators. LINC Consortium. [file jamapsychiatry-e261064-s003.pdf]

Supplemental Online Content: Nonauthor Collaborators
*First name, last name, and suffix (if applicable) are required and will appear in PubMed.

*Group Name(s): LINC Consortium

*First Name and Middle Initial(s)	*Last Name	*Suffix (eg, Jr, III)	Academic Degrees	Institution	Location (city, state/province, country)	Role or Contribution, eg, chair, principal investigator	Group (if more than 1 Group listed in the byline) and/or Subgroup (eg, Steering Committee)
Marianne B. M.	van den Bree			Cardiff University	Cardiff, UK		
George	Kirov			Cardiff University	Cardiff, UK		
Michael J.	Owen			Cardiff University	Cardiff, UK		
James T. R.	Walters			Cardiff University	Cardiff, UK		
Peter A.	Holmans			Cardiff University	Cardiff, UK		
Jane	Lynch			Cardiff University	Cardiff, UK		
Ioanna K.	Katzourou			Cardiff University	Cardiff, UK		
Jack F. G.	Underwood			Cardiff University	Cardiff, UK		
David A.	van Heel			Queen Mary University of London	London, UK		
Sarah	Finer			Queen Mary University of London	London, UK		
Daniel	Stow			Queen Mary University of London	London, UK		
Golam M.	Khandakar			University of Bristol	Bristol, UK		
Nicholas J.	Timpson			University of Bristol	Bristol, UK		
John A. A.	MacLeod			University of Bristol	Bristol, UK		
Julie P.	Clayton			University of Bristol	Bristol, UK		
Ruby S. M.	Tsang			University of Bristol	Bristol, UK		
Jane	Sprackman			University of Bristol	Bristol, UK		
Shahid	Khan			University of Bristol	Bristol, UK		
Inês	Barroso			University of Exeter	Exeter, UK		
Rupert A.	Payne			University of Exeter	Exeter, UK		
Mark	Mon-Williams			University of Leeds	Leeds, UK		
Megan L.	Wood			University of Leeds	Leeds, UK		
Nabila	Ali			University of Leeds	Leeds, UK		
Hilary C.	Martin			Wellcome Sanger Institute	Hinxton, UK		
Thomas	Werge			Institute of Biological Psychiatry	Roskilde, Denmark		
Andrés	Ingason			Institute of Biological Psychiatry	Roskilde, Denmark		
Morteza	Vaez			Institute of Biological Psychiatry	Roskilde, Denmark		

Supplemental Online Content: Nonauthor Collaborators
*First name, last name, and suffix (if applicable) are required and will appear in PubMed.

***First Name and Middle Initial(s)**	***Last Name**	***Suffix (eg, Jr, III)**	Academic Degrees	Institution	Location (city, state/province, country)	Role or Contribution, eg, chair, principal investigator	Group (if more than 1 Group listed in the byline) and/or Subgroup (eg, Steering Committee)
Lam O.	Huang			Institute of Biological Psychiatry	Roskilde, Denmark		
