# Supplementary material for: Recurrent Copy Number Variants and Psychiatric Outcomes in the Context of Polygenic Scores
Source: JAMA Psychiatry. 2026 May 27;83(8):827–36. doi: 10.1001/jamapsychiatry.2026.1064 (PMC13217261; doi:10.1001/jamapsychiatry.2026.1064)
Supplement: Supplement 4. — Data Sharing Statement. [file jamapsychiatry-e261064-s004.pdf]

Data Sharing Statement

Vaez. Recurrent Copy Number Variants and Psychiatric Outcomes in the Context of Polygenic Scores. *JAMA Psychiatry*. Published May 27, 2026. doi:10.1001/jamapsychiatry.2026.1064

Data
Data available: No

Additional Information
Explanation for why data not available: Regarding access to study data (other than sensitive person-level data, which by requirement of the data custodian and Danish legislation cannot be shared) please contact the corresponding author.
